# Prevalence and predictive value of sarcopenia in surgically treated cholangiocarcinoma: a comprehensive review and meta-analysis

**DOI:** 10.3389/fonc.2024.1363843

**Published:** 2024-03-19

**Authors:** Jie He, Yuanyuan Huang, Na Huang, Jiaqing Jiang

**Affiliations:** ^1^ Clinical Medical College of Chengdu Medical College, Chengdu, Sichuan, China; ^2^ Department of Pulmonary and Critical Care Medicine, The First Affiliated Hospital of Chengdu Medical College, Chengdu, Sichuan, China

**Keywords:** sarcopenia, cholangiocarcinoma, prognosis, skeletal muscle mass index, meta-analysis

## Abstract

**Background:**

Sarcopenia, marked by a reduction in skeletal muscle mass and function, is a condition that can manifest in elderly patients with cancer and has been recognized as a possible adverse factor affecting the survival of individuals diagnosed with malignant tumors. This systematic review and meta-analysis aimed to examine the prevalence of sarcopenia in individuals with cholangiocarcinoma while concurrently investigating the potential correlations between the presence of sarcopenia and various critical factors, including survival outcomes and postoperative complications.

**Methods:**

A comprehensive search was conducted across multiple databases, including EMBASE, PubMed, Web of Science, Cochrane Library, and CNKI, employing keywords such as sarcopenia, cholangiocarcinoma, and prognosis. This research explored the prognostic value of sarcopenia on the survival of cholangiocarcinoma. The findings of this meta-analysis were presented using forest plots and a summarized effects model. The Newcastle-Ottawa Scale (NOS) was employed to evaluate the quality of the studies included in the analysis.

**Results:**

A total of 33 articles from five databases were in in the quantitative analysis. A comprehensive meta-analysis revealed that the overall prevalence of sarcopenia among individuals diagnosed with cholangiocarcinoma was43%. Moreover, the analysis revealed a significant and noteworthy correlation between sarcopenia and key clinical parameters such as overall survival (OS), Recurrence-Free Survival (RFS), and Disease-Free Survival (DFS) in patients with cholangiocarcinoma. Subgroup analysis revealed that, when categorized by various ethnicities, diagnostic techniques, and tumor locations, sarcopenia consistently retained its status as a negative predictive factor. Furthermore, sarcopenia has emerged as a risk factor for postoperative complications. All included studies had an NOS score greater than 5, indicating a high quality of evidence.

**Conclusion:**

The results suggest that sarcopenia is significantly related to survival outcomes and postoperative complications in cholangiocarcinoma. Appropriate diagnosis and treatment of sarcopenia should be implemented to improve the prognosis of individuals with cholangiocarcinoma.

**Systematic Review Registration:**

https://www.crd.york.ac.uk/prospero/display_record.php?ID=CRD42023479866, identifier CRD42023479866.

## Introduction

Cholangiocarcinoma, a highly malignant tumor of biliary epithelial cells with poor prognosis, can be categorized into three distinct anatomical regions: intrahepatic, perihilar, and distal bile duct (1). It is imperative to recognize that each of these anatomical subtypes presents distinctive clinical manifestations, demanding tailored therapeutic strategies for optimal patient care (2). The most frequently occurring cancer at the bifurcation of the bile duct is perihilar cholangiocarcinoma (3).In contrast, intrahepatic cholangiocarcinoma is the second most prevalent hepatic malignancy, often marked by delayed diagnosis and fatal outcomes, surpassed only by hepatocellular carcinoma (4). Cholangiocarcinoma accounts for 3% of total gastrointestinal tumors and comprises 10%-15% of all hepatic and biliary tumors (5). In recent decades, the global incidence of cholangiocarcinoma has increased, particularly in Asian nations (6). Presently, the predominant approach for the treatment of most patients with cholangiocarcinoma is surgical resection in conjunction with chemotherapy or chemoradiation (7). Nevertheless, post-surgery, chemotherapy, and chemoradiation frequently give rise to a range of complications and adverse reactions, thereby contributing to an unfavorable prognosis for patients. In addition to factors associated with tumors, several modifiable elements can be adjusted or improved, and these factors significantly influence the prognosis of these individuals (8, 9). Consequently, the identification of these factors will aid healthcare professionals in making informed treatment decisions aimed at enhancing the prognosis of individuals with cholangiocarcinoma.

Recently, there has been growing focus on the impact of alterations in body composition on the prognosis of individuals diagnosed with cholangiocarcinoma. As individuals age, these alterations in body composition primarily entail a reduction in muscle mass, coupled with a simultaneous increase in adipose tissue (10). Sarcopenia is a pivotal clinical aspect of malnutrition linked to cancer (11). Cancer-related malnutrition is a multifaceted syndrome characterized by a gradual decline in skeletal muscle mass, strength, and functionality (12). Computed tomography (CT) has become a valuable tool for tumor imaging and assessing the effectiveness of treatments in cancer patients, and it can also be used to evaluate skeletal muscle mass (13, 14). The evaluation of Skeletal Muscle Index (SMI) through CT demonstrates a significant association with overall muscle mass and represents the prevailing method for characterizing sarcopenia (15). A systematic review of 18 articles encompassing 2929 individuals with cholangiocarcinoma revealed that the prevalence of postoperative sarcopenia among these individuals was 48.4%. Moreover, sarcopenia exhibited a substantial and independent association with overall survival (16). Additionally, in the past two years, multiple studies have assessed the predictive significance of sarcopenia in individuals with cholangiocarcinoma. However, the outcomes of these investigations have exhibited variations and controversies.

Hence, a systematic review and meta-analysis were performed to ascertain the overall prevalence and evaluate the predictive significance of sarcopenia in individuals with cholangiocarcinoma. The aim was to equip clinicians with ample data to facilitate informed decisions aimed at enhancing the prognosis of such individuals.

## Methodology

This systematic review followed the principles outlined in the Preferred Reporting Items for Systematic Reviews and Meta-Analyses (PRISMA) (17). This meta-analysis was registered in PROSPERO (registration number: CRD42023479866).

### Search strategy

Two researchers independently searched for observational studies in specified electronic databases, spanning from the inception of the databases up to November 2023. The PubMed, EMBASE, Cochrane Library, Web of Science, and CNKI databases were used. The search used a combination of MeSH (Medical Subject Headings) terms and specific free words, including sarcopenia, body composition, low skeletal muscle mass, cholangiocarcinoma, skeletal muscle index, cholangiocellular carcinoma, biliary tract cancer, cholangiocellular cancer, and biliary tract carcinoma. Additionally, to identify further relevant articles, the references of the selected articles and pertinent systematic reviews were scrutinized. Before the final analysis, a comprehensive literature re-search was conducted to ensure that the most recent relevant research was not overlooked. **Supplementary Table 1** illustrates the comprehensive search strategy used for each database.

### Inclusion criteria

The inclusion criteria for the study were defined as follows:

- Participants: Individuals over the age of 18 years with cholangiocarcinoma who had undergone surgical resection with or without neoadjuvant therapy, unrestricted by sex, race, or nationality.
- Exposure: Individuals with sarcopenia or low skeletal muscle mass. The diagnostic criteria and cutoff values for sarcopenia were not restricted. All patients were diagnosed with sarcopenia prior to surgery.
- Outcomes: Prevalence of sarcopenia, overall survival (OS), disease-free survival (DFS), recurrence-free survival (RFS), postoperative complications, duration of hospital stay, and in-hospital mortality. Recurrence-free survival was defined as the time from initial surgical resection to recurrence (18). Disease-free survival was defined as survival without death or disease relapse (19).
- Study Design: Observational studies, such as cross-sectional, case-control, and cohort studies, were included.

### Exclusion criteria

The exclusion criteria were defined as follows:

- Studies without clear diagnostic criteria to assess sarcopenia or low skeletal muscle mass.
- Studies that did not include primary data, such as those exclusively available in the form of conference abstracts, editorials, or commentaries, were excluded.
- In cases where the same patient cohort was featured in multiple publications, preference was given to studies that presented more relevant and appropriate data for our investigation.

### Literature selection

Two researchers conducted an initial independent literature screening. In instances of disagreement between researchers, these disparities were addressed through discussion or by reaching a consensus with a third-party reviewer. The removal of duplicate articles was the first step and was executed using EndNote X9 software, followed by the screening of titles and abstracts. Subsequently, the full text of each article was scrutinized to determine their inclusion in the study.

### Data extraction

Two researchers individually extracted and record data within a predefined, standardized electronic spreadsheet utilizing Microsoft Excel 2019. Any disagreements that arose during this process were resolved through discussion or by reaching a consensus with a third-party reviewer. The following data were systematically retrieved:

- Study attributes, including the first author’s name and year of publication.
- Specifics regarding the study design, setting, and geographical region where the research was conducted.
- Pertinent details concerning the study participants included mean age, total sample size, tumor localization, postoperative complications, and sex.
- The diagnostic criteria employed for assessing sarcopenia or low skeletal muscle mass encompassed the methods, parameters, and designated cut-off values.

### Quality assessment of the literature

Two researchers individually assessed the risk of bias (RoB) in the studies incorporated into the review by employing the Newcastle-Ottawa Scale (NOS) (20). The NOS encompasses three dimensions comprising eight items, with a maximum attainable score of nine points. Studies that received an NOS score greater than seven were categorized as having a low RoB, those scoring between 5 and 7 were classified as demonstrating a moderate risk, and those scoring less than five were deemed to have a high risk. Any discrepancies between the researchers were addressed through discussion or by consultation with a third reviewer.

### Statistical analysis

In this study, the primary outcomes of interest included the prevalence of sarcopenia, OS, DFS, and RFS. The secondary outcomes included postoperative complications, sepsis, and hospital stay. For each specific outcome, the hazard ratios (HRs), prevalence, weighted mean difference (WMD), odds ratios (ORs), and 95% confidence intervals (CIs) were obtained through univariate and/or multivariate analyses conducted in each eligible study. The pooled prevalence, HRs, WMD, ORs, and 95%CIs were quantified. Meta-analyses were executed for each outcome when data from more than two studies were available. Otherwise, a descriptive analysis was performed.

Additionally, a subgroup analysis was executed based on tumor localization, SMI/Psoas Muscle Index (PMI), and ethnicity. The Cochrane’s Q statistic and the degree of heterogeneity were quantified using the I^2^ statistic, with I^2^>=50% and *P*<0.10 indicated significant and substantial heterogeneity. A fixed-effects model was applied in the absence of substantial heterogeneity, whereas a random-effects model was used in the presence of substantial heterogeneity. Sensitivity analysis was carried out by sequential exclusion of every study and subsequent recalculation of the statistics. To assess publication bias, funnel plots were scrutinized or Egger’s test was employed when the number of studies exceeded 10. A two-sided *P*<0.05 was deemed statistically significant. All statistical analyses were performed using Stata v 10.0 (StataCorp, College Station, TX, USA) and R 4.1.3 software (https://www.r-project.org/about.html).

## Results

### Search results

**Figure 1:**
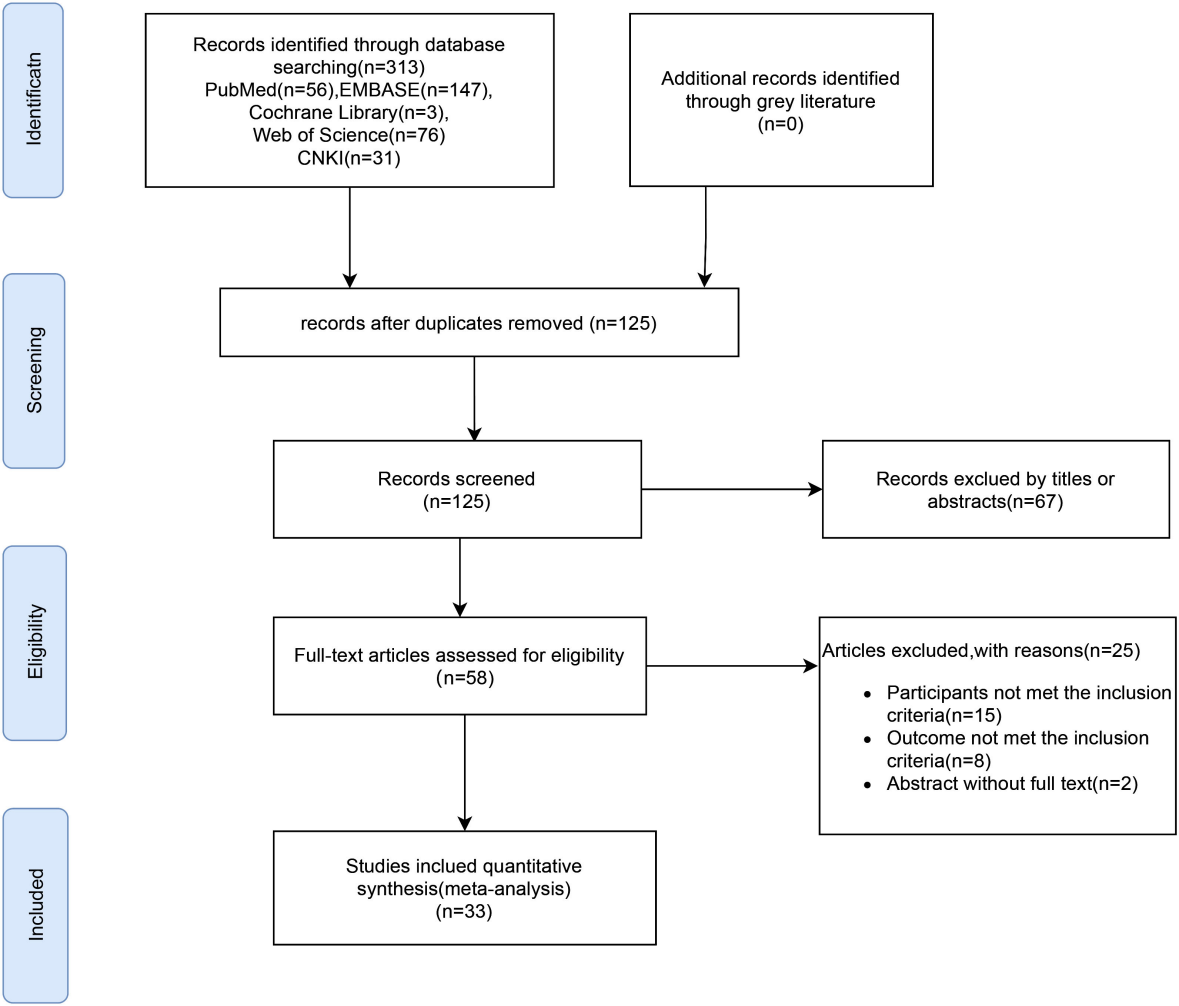
Flow diagram of literature screening.

The selection process for the literature included in this meta-analysis is shown in the flow diagram in **Figure 1**. After an initial search of the electronic databases, 313 studies were obtained. After eliminating duplicate entries, 125 studies were excluded. A review of titles and abstracts led to the exclusion of 67 studies, as they were deemed irrelevant, resulting in 58 studies that were subjected to full-text screening. After full-text screening, 25 studies were eliminated either due to non-compliance with inclusion criteria or due to unavailability of data. Ultimately, 33 article (21–53) were included in the meta-analysis (**Figure 1**).

### Study attributes

In this meta-analysis, two studies were prospective cohort studies; 31 were retrospective cohort studies, and case control studies were among them. Most of this research has been published in the past five years. The cumulative study population included a total of 5173 subjects, with ages ranging from 35 to 89 years. All the studies were included inpatients. Most of the studies have been conducted in Europe and Asia. Most of the literature focuses on intrahepatic cholangiocarcinoma. **Supplementary Table 2** shows the details of the attributes of the included studies.

### Diagnostic criteria for sarcopenia

In this review, the most commonly used method to assess the muscle mass index was SMI calculation using axial CT at the third lumbar vertebra (L3) (n=17). Fifteen studies diagnosed sarcopenia using the PMI. Additionally, one study diagnosed sarcopenia by measuring the total psoas muscle area (TPA).

### Quality evaluation of the incorporated studies

NOS was employed to examine the quality of the studies. 27 studies were considered to be of high quality, and the remainder were of moderate quality. The comprehensive results of quality evaluation is shown in **Supplementary Table 2**.

### Primary outcomes

#### Overall prevalence of sarcopenia in individuals with cholangiocarcinoma

**Figure 2:**
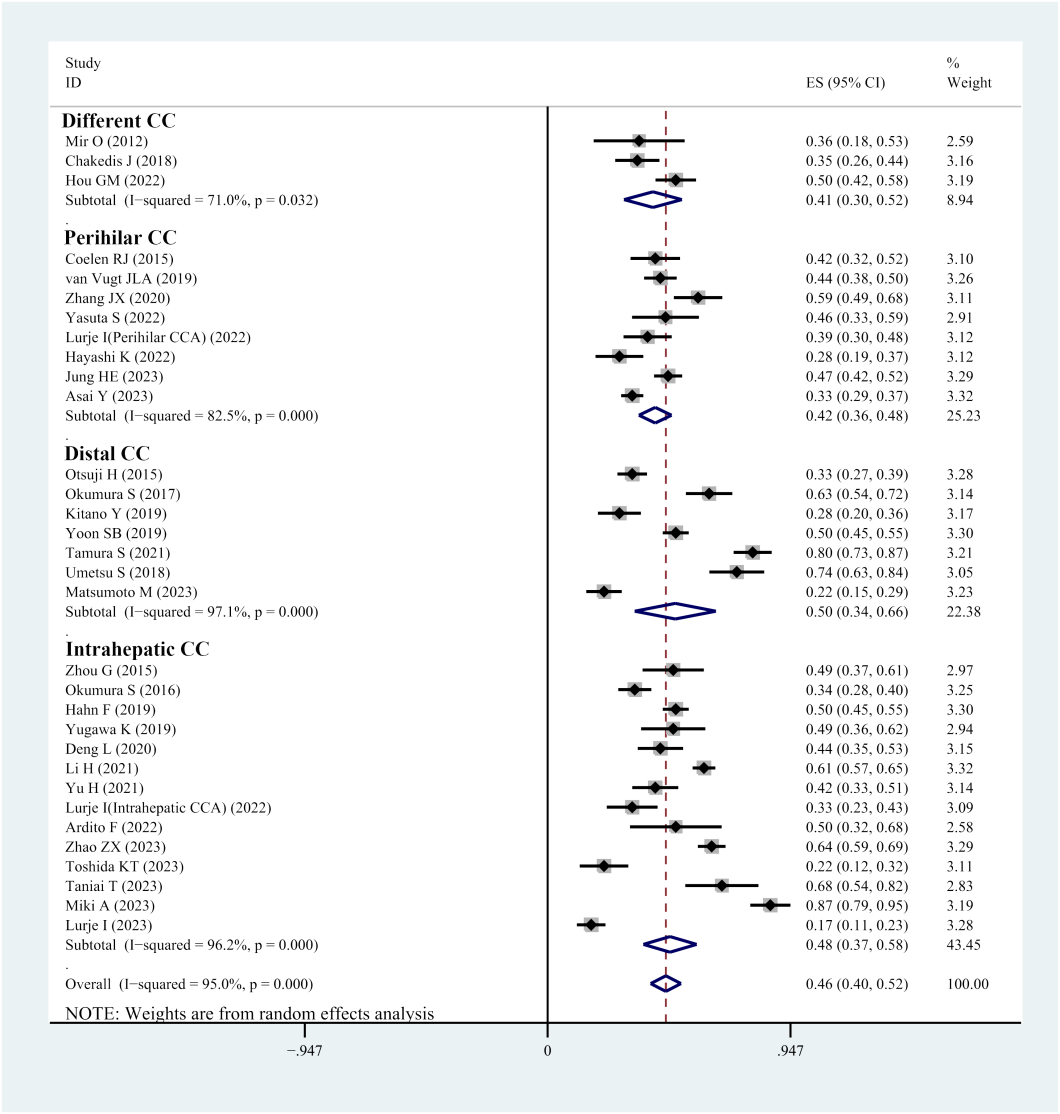
Forest plot of prevalence of sarcopenia in patients with cholangiocarcinoma.

Thirty-two studies reported the prevalence of sarcopenia in individuals with cholangiocarcinoma. The reported prevalence rates in these studies range from 13% to 86%. Heterogeneity tests indicated considerable heterogeneity; hence, a random-effects model was utilized (I^2^ = 95.1%, *P*<0.001). The meta-analysis demonstrated that the overall prevalence of sarcopenia in individuals with cholangiocarcinoma was 46% (95% CI, 40%-52%) (**Figure 2**). Visual analysis using a funnel plot (**Supplementary Figure 1**) and Egger’s test (*P*=0.917) did not reveal any indications of publication bias. Sensitivity analysis demonstrated that no single study considerably affected the overall prevalence of sarcopenia in individuals, underscoring the robustness of the meta-analysis results (**Supplementary Figure 2**).

Subgroup analysis, categorized by the location of cholangiocarcinoma, highlighted that the prevalence of sarcopenia was 42% in individuals with perihilar cholangiocarcinoma, 50% in patients with distal cholangiocarcinoma and 48% in those with intrahepatic cholangiocarcinoma (**Figure 2**).

Further, subgroup analyses were performed based on different skeletal muscle measurement methods. In studies using PMI, the prevalence of sarcopenia was 47%, whereas in studies using SMI, the prevalence of sarcopenia was 46% (**Table 1**).

**Table 1:** Subgroup analyses on the prevalence of sarcopenia and sarcopenic obesity.

Subgroup analysis of prevalence of sarcopenia (n)	ES (95% CI), *P*-value,I^2^ (%),*P*_h_	Subgroup analysis of prevalence of sarcopenic obesity (n)	ES (95% CI), *P*-value,I^2^ (%),*P*_h_
Overall (32)	0.46 (0.40,0.52),<0.0001,95.0%,<0.0001	Overall (5)	0.09 (0.05,0.12),<0.0001,61.2%,<0.0001
Anatomical classification of cholangiocarcinoma			
Intrahepatic CC (14)	0.48 (0.37,0.58),<0.0001,96.2%,<0.0001		
Perihilar CC (8)	0.42 (0.36,0.48),<0.0001,82.5%,<0.0001		
Distal CC (7)	0.50 (0.34,0.66),<0.0001,97.1%,<0.0001		
Different CC (3)	0.41 (0.30,0.52),<0.0001,71.0%,0.032		
Ethnicity			
Asian (23)	0.49 (0.42,0.56), <0.0001,95.5%,<0.0001		
Caucasian (9)	0.38 (0.29,0.47) ,<0.0001, 90.3%,<0.0001		
Test for sarcopenia			
PMI (14)	0.47 (0.40,0.52) ,<0.0001, 94.9%,<0.0001		
SMI (17)	0.46 (0.37,0.55) ,<0.0001, 95.3%,<0.0001		
TPA (1)	NA		

CC, cholangiocarcinoma; PMI, Psoas Muscle Index; SMI, Skeletal Muscle Index; TPA, psoas muscle area; NA, not applicable.

Subgroup analyses based on ethnicity demonstrated that the prevalence of sarcopenia was 38% in Caucasian patients, while it was 46% in Asian patients (**Table 1**).

Five studies reported on the prevalence of sarcopenic obesity in individuals with cholangiocarcinoma. A meta-analysis employing a random-effects model highlighted that the prevalence of sarcopenic obesity in patients was 9% (95% CI, 5%-12%) (**Supplementary Figure 3**).

#### Overall survival

**Figure 3:**
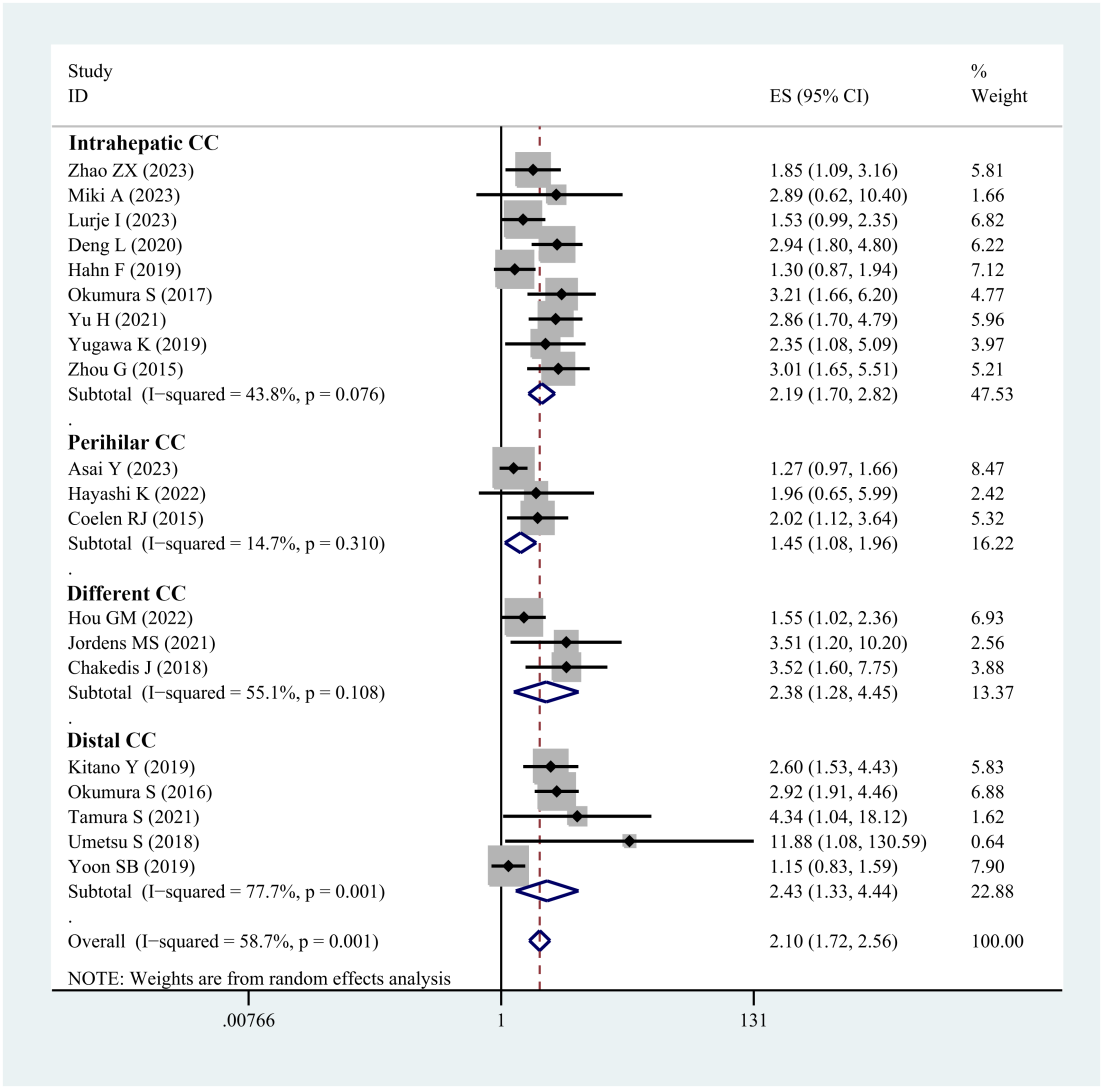
Forest plot of studies evaluating hazard ratios of sarcopenia and the overall survival of cholangiocarcinoma (adjusted hazard ratio values).

HR analysis for OS included 24 studies. Significant heterogeneity was observed, necessitating the use of a random-effects model. The meta-analysis demonstrated a notable link between sarcopenia and reduced OS in both multivariate (HR=2.10; 95% CI, 1.72-2.56, I^2^ = 58.7%, *P*<0.001, 20 studies; **Figure 3**) and univariate analyses (HR=2.04; 95% CI, 1.74-2.40, I^2^ = 0%, *P*<0.001, 10 studies; **Supplementary Figure 4**). Subgroup analyses were carried out based on tumor location, diagnostic method, whether adjuvant chemotherapy was combined, and ethnicity: detailed results are presented in **Table 2** and **Supplementary Table 3**. The funnel plot displayed symmetry, indicating no evidence of publication bias, a conclusion supported by the Egger’s test (*P*=0.183).

**Table 2:** Subgroup analyses in terms of overall survival, disease-free survival and Recurrence-free survival (adjusted HR).

Characteristics	Overall survival				Disease-free survival				Recurrence-free survival			
	n	HR (95%CI)	P	Heterogeneity	n	HR (95%CI)	P	Heterogeneity	n	HR (95%CI)	P	Heterogeneity
Anatomical classification of CC	20	2.10 (1.72,2.56)	<0.001	58.70%	3	2.52 (1.35,4.69)	0.004	74.10%	6	2.33 (1.93,2.82)	<0.001	0.00%
Intrahepatic CC	9	2.19 (1.70,2.82)	0.015	43.80%	2	3.45 (2.23,5.34)	<0.001	0.00%				
Perihilar CC	3	1.45 (1.08,1.96)	<0.001	14.70%	1	NA	NA	NA				
Distal CC	5	2.43 (1.33,4.44)	0.004	77.70%								
Different CC	3	2.38 (1.28,4.45)	0.006	55.10%								
Ethnicity												
Asian	15	2.19 (1.72,2.80)	<0.001	63.40%								
Caucasian	5	1.87 (1.31,2.66)	0.001	45.40%								
Test for sarcopenia												
PMI	12	2.18 (1.64,2.89)	<0.001	59.20%					4	2.29 (1.76,2.99)	<0.001	20.40%
SMI	8	2.05 (1.51,2.77)	<0.001	63.10%					2	2.38 (1.82,3.10)	<0.001	0.00%
Adjuvant chemotherapy												
Yes	2	2.99 (1.72,5.21)	<0.001	0.00%								
No	6	2.04 (1.43,2.89)	<0.001	67.50%								
Any	12	2.08 (1.58,2.74)	<0.001	59.70%								

CC, cholangiocarcinoma; PMI, Psoas Muscle Index; SMI, Skeletal Muscle Index; NA, not applicable.

**Figure 4:**
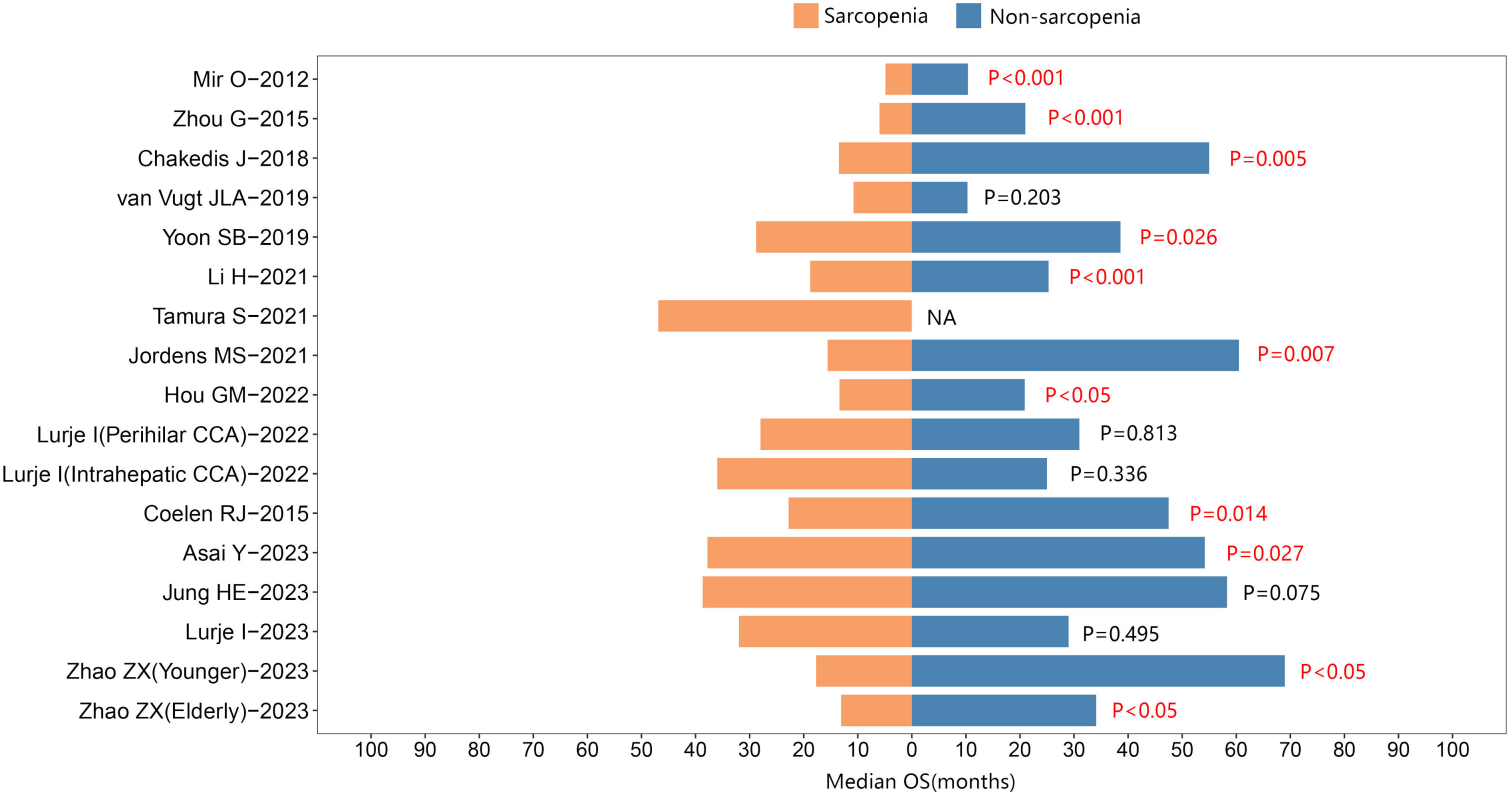
The median overall survival between sarcopenic and nonsarcopenic patients with cholangiocarcinoma.

Furthermore, 17 studies reported the median survival times. Among them, 11 studies indicated that individuals with sarcopenia exhibited shorter median survival times than those without sarcopenia in cholangiocarcinoma. See **Figure 4**.

#### Recurrence-free survival

Ten studies were included in the HR analysis for RFS. The meta-analysis, employing a fixed-effects model, demonstrated a significant association between sarcopenia and adverse RFS in both multivariate (HR= 2.33, 95% CI 1.93-2.82, I^2^ = 0%, *P*<0.001, 6 studies; **Supplementary Figure 5**) and univariate analyses (HR= 1.88, 95% CI 1.55-2.28, I^2^ = 0%, *P*<0.001, 9 studies; **Supplementary Figure 6**). The outcomes of subgroup analyses are presented in the **Table 2** and **Supplementary Table 3**.

**Figure 5:**
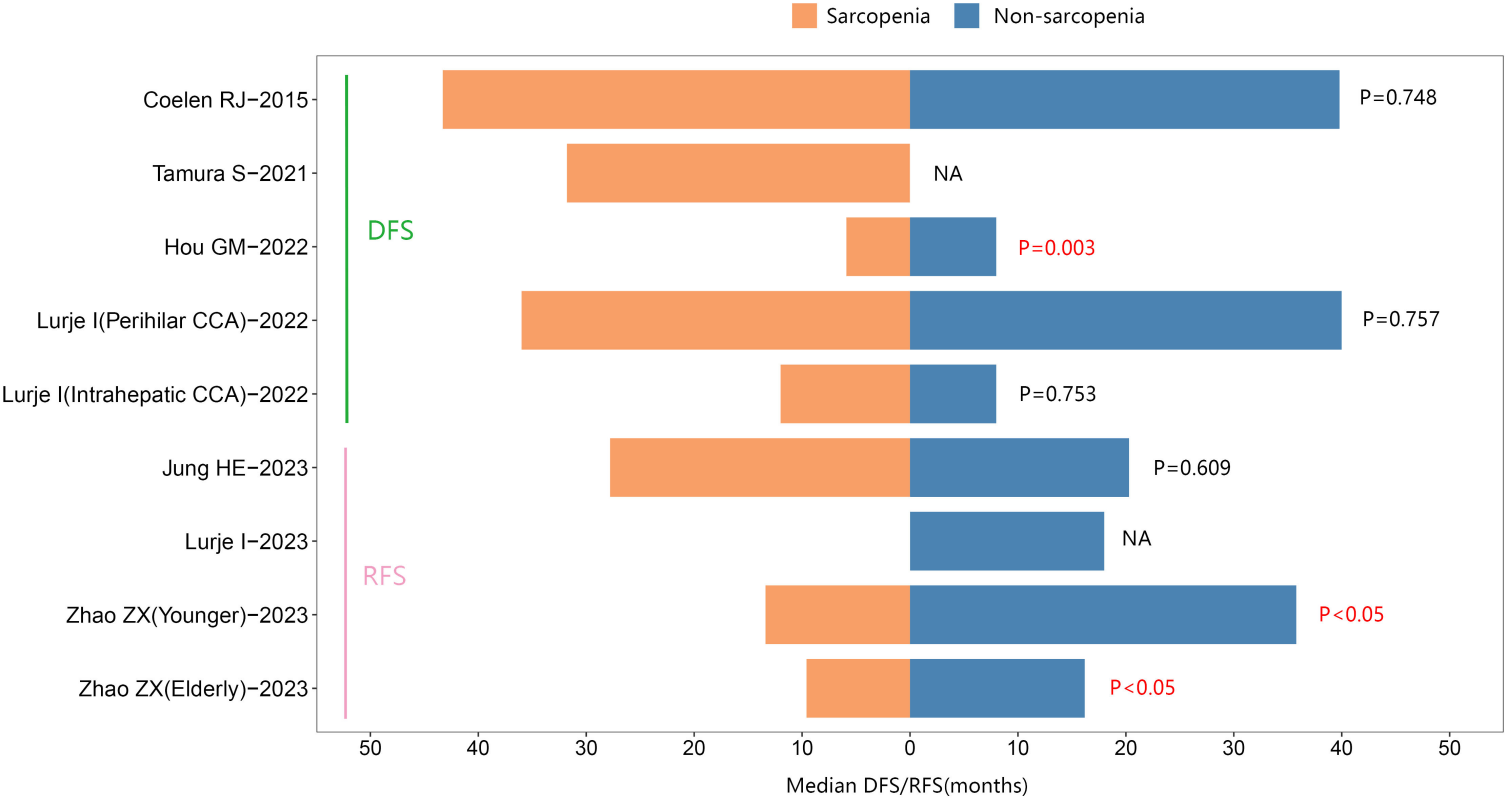
The median Disease-Free Survival/Recurrence-Free survival between sarcopenic and nonsarcopenic patients with cholangiocarcinoma.

In addition, four studies reported median RFS. Of these, two studies suggested that individuals with sarcopenia exhibited a shorter median RFS than those without sarcopenia in cholangiocarcinoma. See **Figure 5**.

#### Disease-free survival

Four studies were included in the HR analysis of DFS. The meta-analysis carried out using a random-effects model, revealed a notable association between sarcopenia and poorer DFS in multivariate analysis (HR= 2.14, 95% CI 1.62-2.83, I^2^ = 74.1%, *P*<0.001, three studies; **Supplementary Figure 7**). Additionally, the same relationship was noticed in univariate analysis utilizing a fixed-effects model (HR= 2.20, 95% CI 1.68-2.88, I^2^ = 37.8%, *P*<0.001, four studies; **Supplementary Figure 8**). The findings of subgroup analyses are shown in **Table 2** and **Supplementary Table 3**.

Additionally, five studies reported the median DFS. One study suggested that individuals with sarcopenia had a shorter median DFS than those without sarcopenia in cholangiocarcinoma. See **Figure 5.**

### Secondary outcomes

#### Postoperative complications

**Figure 6:**
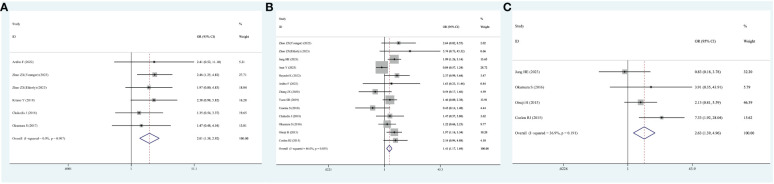
Forest plot of odds ratios for postoperative complications in sarcopenic patients compared with non-sarcopenic patients with cholangiocarcinoma. **(A)** Unspecified complication; **(B)** severe complication; **(C)** sepsis.

Six studies reported on the number of patients who developed postoperative complications. Given the lack of significant heterogeneity in the test results, a fixed-effects model was employed. The meta-analysis highlighted that individual with cholangiocarcinoma who had sarcopenia exhibited a heightened risk of postoperative death relative to those without sarcopenia (OR=2.01; 95% CI, 1.38-2.92, I^2^ = 0%, *P*<0.001, **Figure 6A**).

Thirteen studies reported the number of patients who developed serious postoperative complications. Again, because of the non-significant heterogeneity, a fixed-effects model was employed. The meta-analysis highlighted that cholangiocarcinoma patients with sarcopenia exhibited a heightened risk of serious postoperative complications relative to those without sarcopenia (OR=1.41; 95% CI, 1.17-1.69, I^2^ = 46%, *P*<0.001, **Figure 6B**).

Notably, four studies specifically reported the number of patients who developed postoperative sepsis. Because no significant heterogeneity was observed, a fixed-effects model was used. The meta-analysis demonstrated that cholangiocarcinoma patients with sarcopenia exhibited a heightened risk of developing sepsis after surgery relative to those without sarcopenia (OR=2.63; 95% CI, 1.39-4.96, I^2^ = 36.9%, *P*=0.003, **Figure 6C**).

#### Postoperative mortality

Ten studies reported the number of postoperative deaths among the included patients, and as heterogeneity was not significant, a fixed-effects model was utilized. The meta-analysis found that the risk of postoperative mortality was higher in individuals with cholangiocarcinoma who had sarcopenia than in those without sarcopenia (OR=2.84; 95% CI, 1.90-4.26, I^2^ = 0%, *P*<0.001, **Supplementary Figure 9**).

#### Length of hospital stay

Eight studies provided data on the length of hospital stay of the included patients. Owing to the significant heterogeneity, a random-effects model was used. The meta-analysis found that cholangiocarcinoma patients with sarcopenia exhibited a longer hospital stay (WMD=2.37; 95% CI, 1.62-2.83, I^2^ = 50.1%, *P*=0.003, **Supplementary Figure 10**).

## Discussion

Some patients diagnosed with cholangiocarcinoma may experience challenges such as reduced food intake resulting from pain or prolonged bed rest after surgery, putting them at a relatively high risk of malnutrition (54). Therefore, it is crucial to find a reliable indicator to determine the nutritional status of individuals with cholangiocarcinoma and provide appropriate nutritional support. Traditional Body Mass Index (BMI) cannot accurately reflect nutritional status and body composition and has certain limitations (55). Other indices, including Patient-Generated Subjective Global Assessment (PG-SGA), Malnutrition Screening Tool (MST), and Nutritional Risk Screening (NRS), have been used clinically to determine the nutritional level of individuals with cancer (56). Nevertheless, the predictive value of these indicators is limited, and they have not been widely applied in clinical practice. Consequently, there is a need to identify an indicator that to accurately predicts the malnutrition status of individuals with cholangiocarcinoma. Sarcopenia has recently emerged as a promising new nutritional marker owing to its strong predictive capabilities. Sarcopenia is characterized by the infiltration of connective tissue and fat into muscle tissue, reduction in the number of type 1 and type 2 fibers, and decrease in motor units (57). As a result, sarcopenia has garnered attention as a pivotal indicator for assessing nutritional status, particularly in cancer patients.

This research examined the overall prevalence and prognostic significance of sarcopenia in individuals with cholangiocarcinoma. The findings indicate that the overall prevalence of sarcopenia in individuals with cholangiocarcinoma is 46%, with a higher proportion of patients with distal cholangiocarcinoma developing sarcopenia than those with intrahepatic cholangiocarcinoma (50% vs. 48%). Sarcopenia is significantly associated with poor OS, RFS, and DFS, increased risk of postoperative death, postoperative complications, and longer hospital stays.

This study found that over 40 percent of adult patients with cholangiocarcinoma were affected by sarcopenia, and nearly one in ten patients present with sarcopenic obesity. The prevalence of sarcopenia in individuals with distal cholangiocarcinoma is slightly elevated relative to the other two types of cholangiocarcinoma. Embryologically, intrahepatic cholangiocytes arise from bipotent hepatoblasts, and extrahepatic cholangiocytes share an embryologic origin with the ventral pancreas (58). Patients with distal cholangiocarcinoma are more prone to malignant obstruction of the biliary tract, resulting in a worse quality of life. Surgery for distal cholangiocarcinoma, like all peripancreatic malignancies, requires a pancreaticoduodenectomy, a procedure that is more traumatic and associated with significant physical consumption (59). It is worth to mention that the neoadjuvant chemotherapy may have impact on the prevalence of sarcopenia. Taniai et al. (40) reported that there tended to be a lack of adjuvant chemotherapy in sarcopenia group (46% vs. 77%). This phenomenon illustrates that patients with sarcopenia are generally less willing to undergo neoadjuvant chemotherapy than those without sarcopenia. During chemotherapy, patients with cancer may also experience muscle wasting, which can be attributed to factors such as reduced food intake, decreased physical activity due to fatigue, direct effects of chemotherapy on muscles, and poor absorption due to mucositis or anorexia (60). However, the role of adjuvant chemotherapy in patients with resected bile duct cancer is controversial. There are few reports on the impact of neoadjuvant therapy in patients with cholangiocarcinoma, although the efficacy of neoadjuvant therapy followed by surgery for “unresectable” locally advanced cholangiocarcinoma has been reported (61). Moreover, although some studies have reported the incidence of sarcopenia in patients with cholangiocarcinoma, the included patient population was limited to only a subset that received neoadjuvant chemotherapy. Based on available data, we could not determine whether neoadjuvant chemotherapy had a negative impact on the development of sarcopenia. At present, the precise mechanism underlying sarcopenia in individuals diagnosed with cholangiocarcinoma remains unclear. This condition may be attributed to elevated levels of systemic inflammatory markers and insulin resistance in patients with cancer. These factors can potentially disrupt the normal metabolism of fat and result in a reduction in muscle mass, consequently contributing to the development of sarcopenia (62). Conversely, an intricate dynamic involving increased nutritional requirements and decreased intake, which can result from tumor invasion and lead to malnutrition in cancer patients, is also a contributing factor to the development of sarcopenia (63). The effect of sarcopenia on the prognosis of individuals with gastrointestinal tumors has been the focus of most recent studies, mainly on individuals with pancreatic or colorectal cancer who have higher rates of sarcopenia. According to Pamoukdjian et al., the overall prevalence of sarcopenia among cancer patients is 40% (64). The overall prevalence of sarcopenia in cholangiocarcinoma patients included in this research was higher than the average, and sarcopenia may lead to adverse outcomes in the cholangiocarcinoma patient population. Hence, healthcare providers should prioritize screening for sarcopenia in patients with cholangiocarcinoma as a routine aspect of future clinical practice.

The term “sarcopenia” essentially denotes a deficiency in muscle mass. Nevertheless, certain international bodies assert that aside from muscle mass loss, the diagnostic criteria for sarcopenia should encompass a decrease in muscle strength and/or diminished physical performance (65). Although this diagnostic criterion has garnered widespread recognition within the field of geriatric medicine, cancer research still necessitates the evaluation of muscle mass as a fundamental parameter. In our view, it is not rigorous for most studies to define sarcopenia solely by using SMI or PMI. While CT scans are advocated as the gold standard for estimating muscle mass, it is important to note that CT scans are unable to assess muscle strength directly. This study observed that the diagnosis of sarcopenia in most of the studies incorporated in this review relied on SMI, which was assessed by measuring the muscle area on axial CT scans at the L3 level. Nevertheless, a minority of studies have posited that, especially in patients with gastrointestinal tumors, skeletal muscle strength and/or physical function could better predict the predictive significance of cancer-related sarcopenia (66, 67). Consequently, the inclusion of muscle strength and physical function in the future diagnosis of sarcopenia in individuals with cholangiocarcinoma should be validated in further prospective cohort studies.

According to the findings of the meta-analysis, the forest plots demonstrated that sarcopenia can predict worse OS, RFS, and DFS. Moreover, according to the outcomes of subgroup analysis, sarcopenia is still notably linked to unfavorable OS across various populations, tumor localization, and even diverse sarcopenia measurement methods. In the subgroup analysis of patients who received only surgical treatment, sarcopenia was not significantly associated with prognosis. The high heterogeneity may be partly due to the clinical baseline imbalance of various subgroups and the limited number of studies. The high heterogeneity observed in the I^2^ value of this subgroup suggests that the pooled results may deviate from the actual results, and more research is required to validate this conclusion. Most studies show that individuals with sarcopenia have a shorter median OS than to those without sarcopenia. A few studies have indicated that individuals with sarcopenia have shorter median RFS and DFS than those without sarcopenia. Therefore, caution is warranted when interpreting the link between sarcopenia and RFS/DFS, and further investigation is essential. Sarcopenia has also been proven to be a risk factor for postoperative complications, particularly serious complications. Sarcopenia is intricately linked to the occurrence of postoperative sepsis, and patients with sarcopenia seem to have longer hospital stays after surgery than non-sarcopenic patients. When examining the underlying causes, it is evident that patients with sarcopenia are often in a state of heightened inflammation. This inflammatory state leads to the production and secretion of various pro-inflammatory cytokines, including tumor necrosis factor-alpha, interleukin-6, and C-reactive protein, etc. (43, 68, 69). These pro-inflammatory cytokines play a role in immunomodulation and can have detrimental effects on the immune response to surgical stress. They increase the risk of surgical site infections, hinder wound healing, and consequently elevate the risk of postoperative complications. An investigation of extrahepatic cholangiocarcinoma revealed that sarcopenic patients displayed a significantly lower count of CD8+ tumor-infiltrating lymphocytes (TILs) within the tumor than non-sarcopenic patients, indicating that sarcopenia affects not only systemic inflammation but also the local immune system (31). Moreover, research has highlighted the association between sarcopenia and insulin resistance, which can also impede the appropriate response to surgical stress and contribute to various complications (70). Thus, healthcare providers should focus on preoperative screening for sarcopenia, implement timely interventions, and reduce the incidence of postoperative complications.

Some of the studies included in this study reported that patients received neoadjuvant chemotherapy in addition to surgical treatment. Thus, the possibility that sarcopenia could lead to an increase in postoperative chemotherapy toxicity should be considered. Currently, postoperative adjuvant chemotherapy is regarded as a conventional treatment approach, given its potential to enhance patient survival (71). Currently, chemotherapy doses are typically calculated using the body surface area, which is derived from the height and weight of the patient, without considering variations in body composition. It is widely acknowledged that as individuals age, their body composition undergoes alterations, characterized by a decline in muscle mass and elevation in fat mass (72). In certain cases, individuals may even develop sarcopenic obesity, which could lead to an actual chemotherapy dosage that is too high owing to a high body surface area. An overdose of chemotherapeutic drugs can elevate chemotherapy toxicity, decrease the effectiveness of chemotherapy, increase the risk of sepsis, and severely affect the prognosis of patients with cholangiocarcinoma. Hence, clinicians should underscore the significance of considering sarcopenia when evaluating chemotherapy toxicity in cancer patients during clinical practice, given that sarcopenia reflects the nutritional and immune status of the patient (43). Among elderly patients with pancreatic cancer, sarcopenia can predict the poor success rate of adjuvant chemotherapy (73).Sarcopenia is a significant predictor of dose-limiting toxicity in patients with esophago-gastric cancer undergoing neo-adjuvant chemotherapy (74). We hypothesized that SMI/PMI would be good prognostic predictors for cholangiocarcinoma patients receiving neoadjuvant chemotherapy. Unfortunately, in this meta-analysis, no study explicitly addressed the influence of sarcopenia on chemotherapy toxicity. Hence, additional prospective studies are necessary to delve deeper into the influence of sarcopenia on chemotherapy toxicity across various cholangiocarcinoma subtypes.

### Strengths and limitations

Previous studies have published related studies. Suorv et al. (16) performed a meta-analysis to examine the influence of sarcopenia on the long-term survival of individuals with cholangiocarcinoma. However, their analysis incorporated only 18 studies, with a research cut-off date of up to 2021. Over the subsequent two years, several new studies have been compiled and included in our meta-analysis. Surov et al. (75) also conducted a meta-analysis to analyze the prevalence of sarcopenia on staging computed tomography in patients with different malignant solid tumors. Subgroup analysis for the types of cholangiocarcinoma was not performed in their study. The present study has several strengths (1): it encompasses the largest number of studies and sample size, with a total of 33 included articles; (2) the analysis also incorporated the latest literature published up to 2023; and (3) this study provided a more comprehensive analysis than before. Subgroup analyses were conducted based on various cholangiocarcinoma types, and a deeper investigation into the relationship between OS and sarcopenia was performed. Additionally, previous studies did not report on postoperative complications and the length of hospital stay for sarcopenic patients, which were thoroughly assessed in this study. As a result, this research provides the most recent and comprehensive body of evidence to elucidate the effect of sarcopenia on the prognosis of patients with cholangiocarcinoma.

Nevertheless, it is essential to acknowledge the limitations of the present study. First, most of the included studies belonged to the retrospective cohort category, and certain subgroups had relatively small sample sizes, which might have led to an underestimation of the effect of sarcopenia on individuals with cholangiocarcinoma. Second, most studies concerned intrahepatic cholangiocarcinoma, with relatively little data on other locations. The prognosis of cholangiocarcinoma can differ according to its location. Third, there was variability in the diagnostic criteria and cutoff values for sarcopenia among the studies, which could potentially influence the outcomes. Fourth, the restricted number of included studies led to the inability to subject certain results to a meta-analysis, and they were presented graphically. Fifth, the treatment for perihilar cholangiocarcinoma is a highly invasive surgery. Biliary drainage, which can prolong preoperative waiting time (PWT), is often required before surgery. Percutaneous transhepatic biliary external drainage may be the preferred preoperative drainage method for hilar cholangiocarcinoma because of the low incidence of cholangitis and pancreatitis (76). Skeletal muscle mass may change during PWT, which could influence on surgical outcomes of perihilar cholangiocarcinoma. Zhang et al. (49) suggested that sarcopenia was an important predictor of poor OS after percutaneous transhepatic biliary drainage for patients with perihilar cholangiocarcinoma-related obstructive jaundice. Nevertheless, in the present meta-analysis, some included patients were treated with biliary drainage. We could not determine whether sarcopenia is associated with biliary drainage.

### Clinical implications

Overall, the findings of this research affirm the perspective of previous studies indicating that radiologists should provide information regarding tumor staging as well as body composition. This is neither complex nor requires additional analysis, as muscle mass can be assessed during the staging process using CT scans. As mentioned previously, body composition and muscle mass can be affected. Existing research has demonstrated that preoperative exercise and nutritional support programs can mitigate sarcopenia and enhance the likelihood of reduced postoperative complications in surgical patients (77). Similarly, consuming carbohydrate and branched-chain amino acid snacks in the evening can enhance the nutritional status of individuals undergoing liver resection (78). This study suggests that special nutrition and exercise programs for patients with cholangiocarcinoma could also improve body composition, thereby preventing the occurrence of adverse events related to sarcopenia.

## Conclusion

In summary, sarcopenia is very common in cholangiocarcinoma, is a strong prognostic predictor post-surgery, and is closely related to postoperative complications and length of hospital stay. Sarcopenia should be included in the routine postoperative assessment of individuals with cholangiocarcinoma, which may help clinicians adjust their treatment strategies and promptly provide appropriate nutritional support. Future studies, characterized by larger sample sizes and a prospective design, are required to delve deeper into the association between sarcopenia, chemotherapy toxicity, RFS, and DFS in cholangiocarcinoma.

## Data availability statement

The original contributions presented in the study are included in the article/**Supplementary Material**. Further inquiries can be directed to the corresponding author.

## Author contributions

JH: Conceptualization, Data curation, Formal analysis, Funding acquisition, Investigation, Methodology, Project administration, Resources, Software, Supervision, Validation, Visualization, Writing – original draft, Writing – review & editing. YH: Software, Writing – review & editing. NH: Methodology, Software, Writing – review & editing. JJ: Formal analysis, Software, Writing – review & editing.

## Supplementary material

Supplementary Figure 1 Funnel plot for prevalence of sarcopenia in patients with cholangiocarcinoma.

Supplementary Figure 2 Sensitivity analysis for prevalence of sarcopenia in patients with cholangiocarcinoma.

Supplementary Figure 3 Forest plot of prevalence of sarcopenic obesity in patients with cholangiocarcinoma.

Supplementary Figure 4 Forest plot of studies evaluating hazard ratios of sarcopenia and the overall survival of cholangiocarcinoma (unadjusted hazard ratio values).

Supplementary Figure 5 Forest plot of studies evaluating hazard ratios of sarcopenia and the Recurrence-Free survival of cholangiocarcinoma (adjusted hazard ratio values).

Supplementary Figure 6 Forest plot of studies evaluating hazard ratios of sarcopenia and the Recurrence-Free survival of cholangiocarcinoma (unadjusted hazard ratio values).

Supplementary Figure 7 Forest plot of studies evaluating hazard ratios of sarcopenia and the Disease-Free survival of cholangiocarcinoma (adjusted hazard ratio values).

Supplementary Figure 8 Forest plot of studies evaluating hazard ratios of sarcopenia and the Disease-Free survival of cholangiocarcinoma (unadjusted hazard ratio values).

Supplementary Figure 9 Forest plot of odds ratios for mortality associated with surgery in sarcopenic patients compared with non-sarcopenic patients with cholangiocarcinoma.

Supplementary Figure 10 The length of hospital stay between sarcopenic and nonsarcopenic patients with cholangiocarcinoma.
