# Supplementary figures and images for: Prevalence and predictive value of sarcopenia in surgically treated cholangiocarcinoma: a comprehensive review and meta-analysis
Source: Front Oncol. 2024 Mar 19;14:1363843. doi: 10.3389/fonc.2024.1363843 (PMC10989063; doi:10.3389/fonc.2024.1363843)

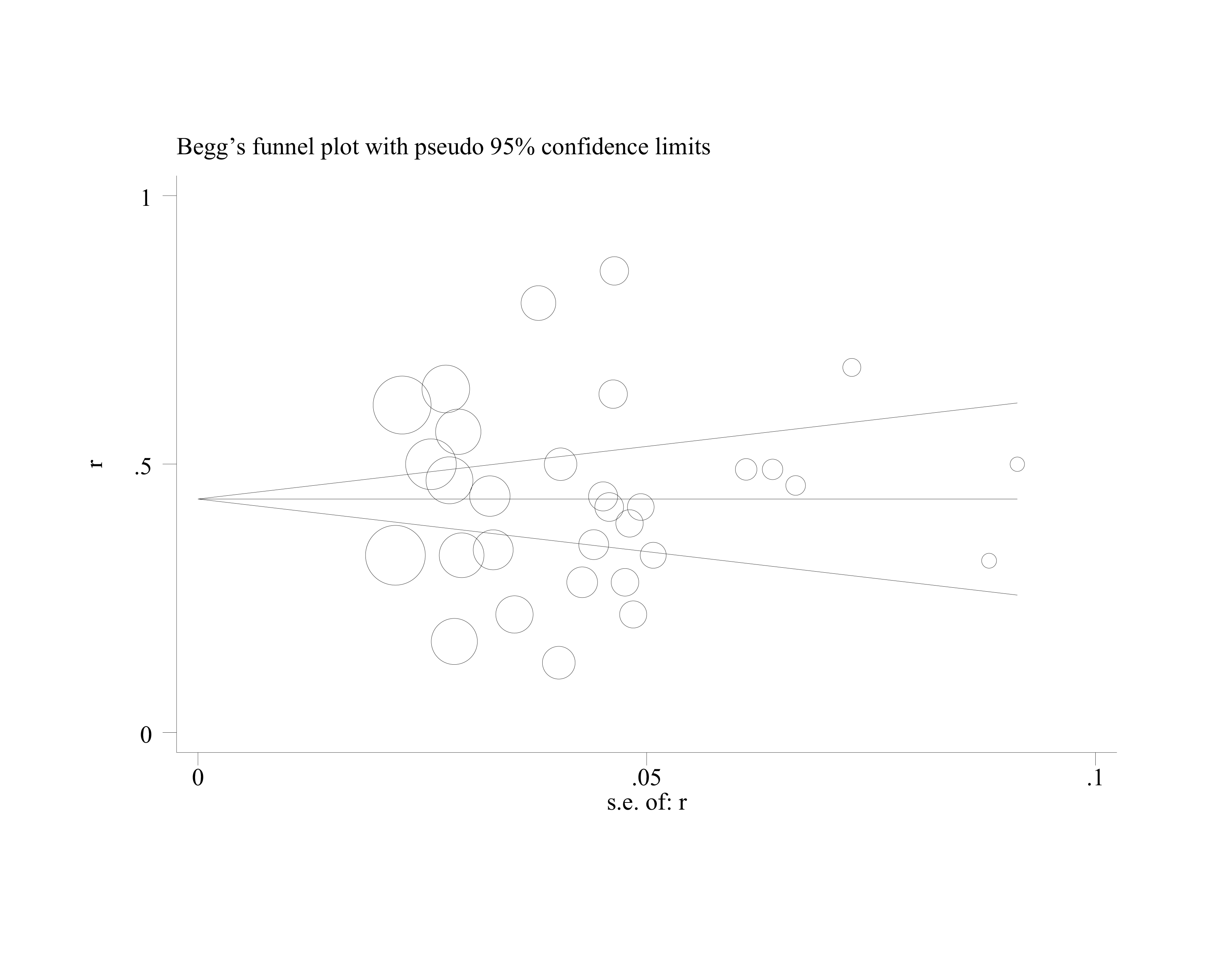

Supplement: Supplementary Figure 1 — Funnel plot for prevalence of sarcopenia in patients with cholangiocarcinoma. [file Image_1.tif]

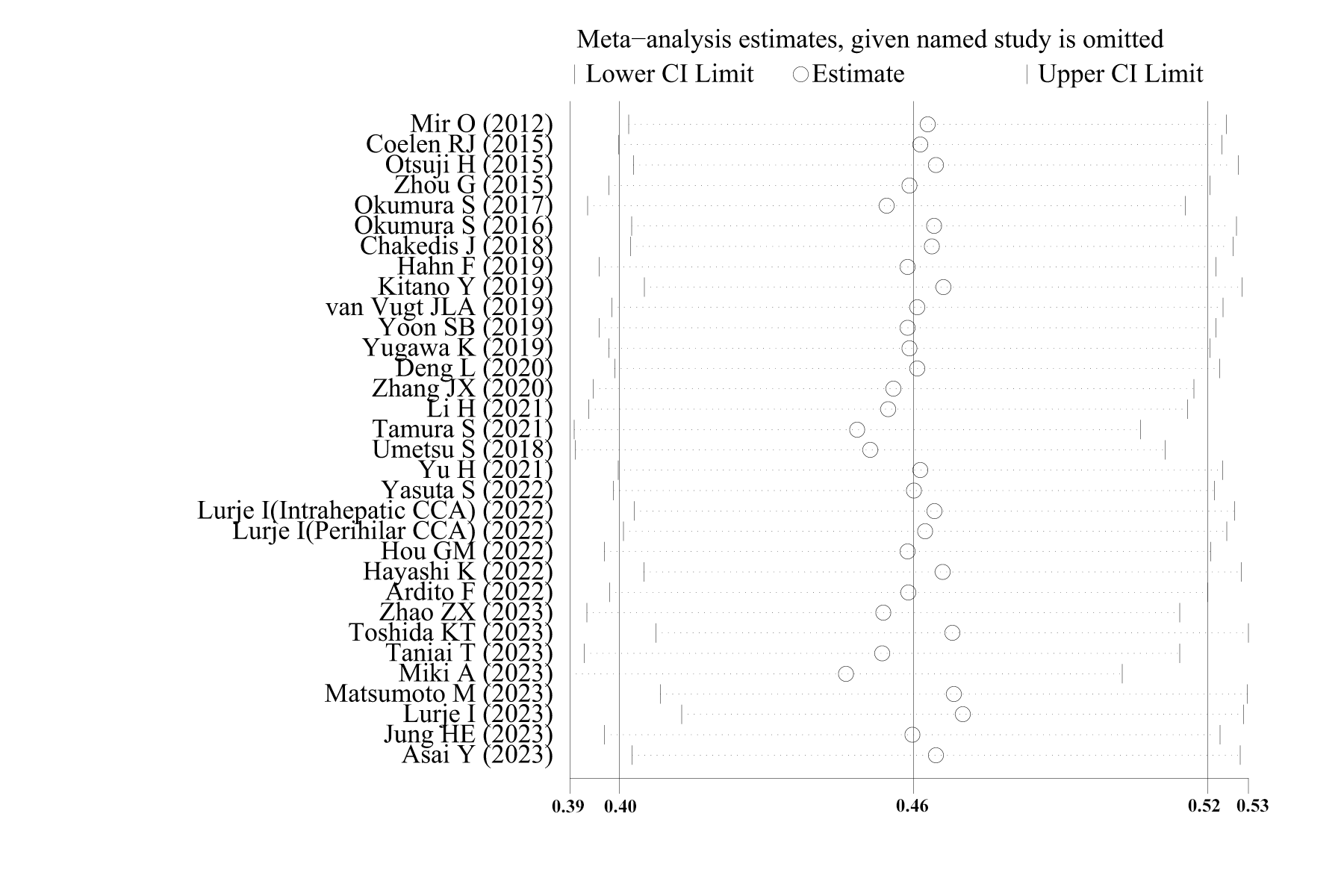

Supplement: Supplementary Figure 2 — Sensitivity analysis for prevalence of sarcopenia in patients with cholangiocarcinoma. [file Image_2.tif]

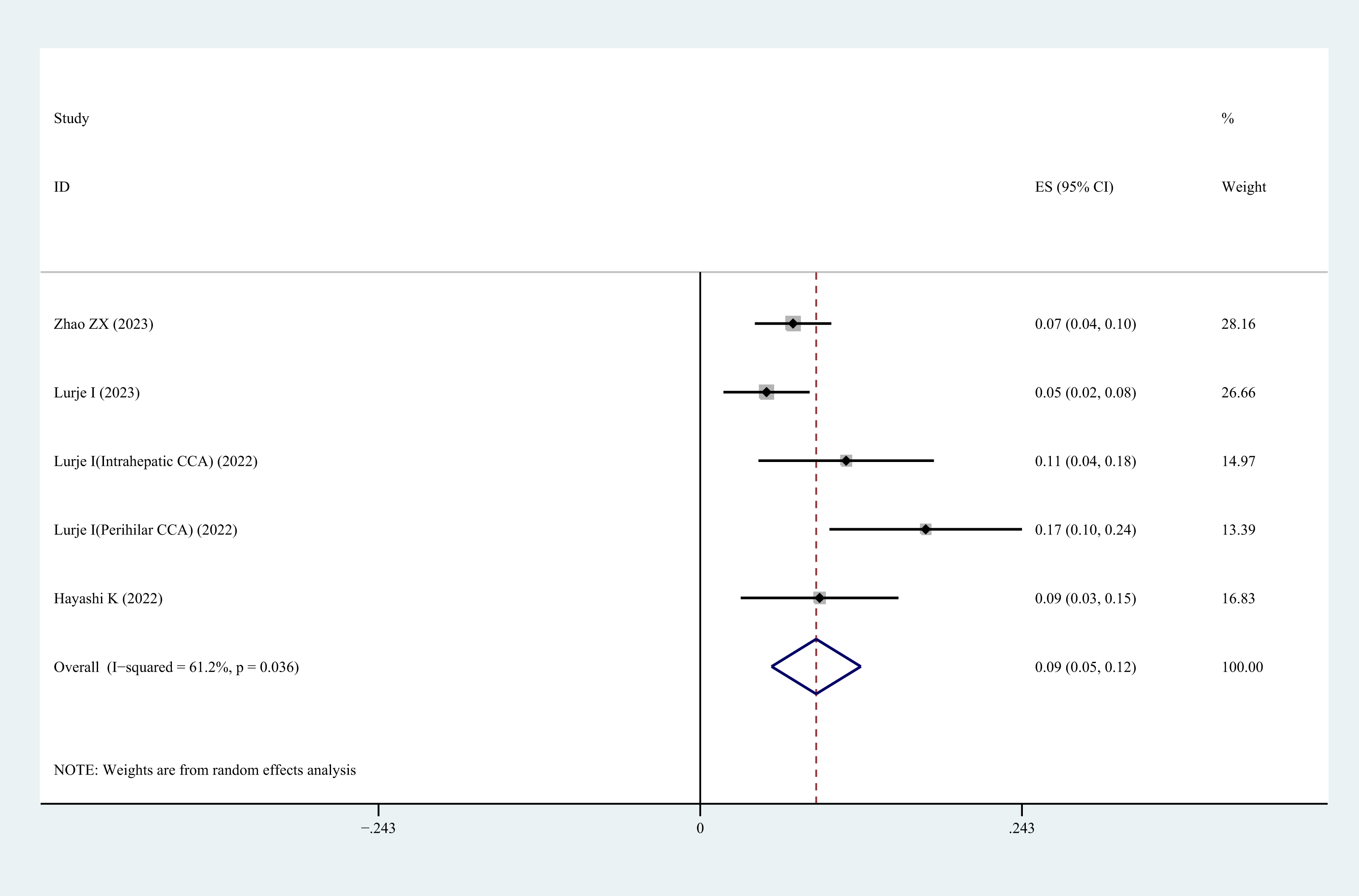

Supplement: Supplementary Figure 3 — Forest plot of prevalence of sarcopenic obesity in patients with cholangiocarcinoma. [file Image_3.tif]

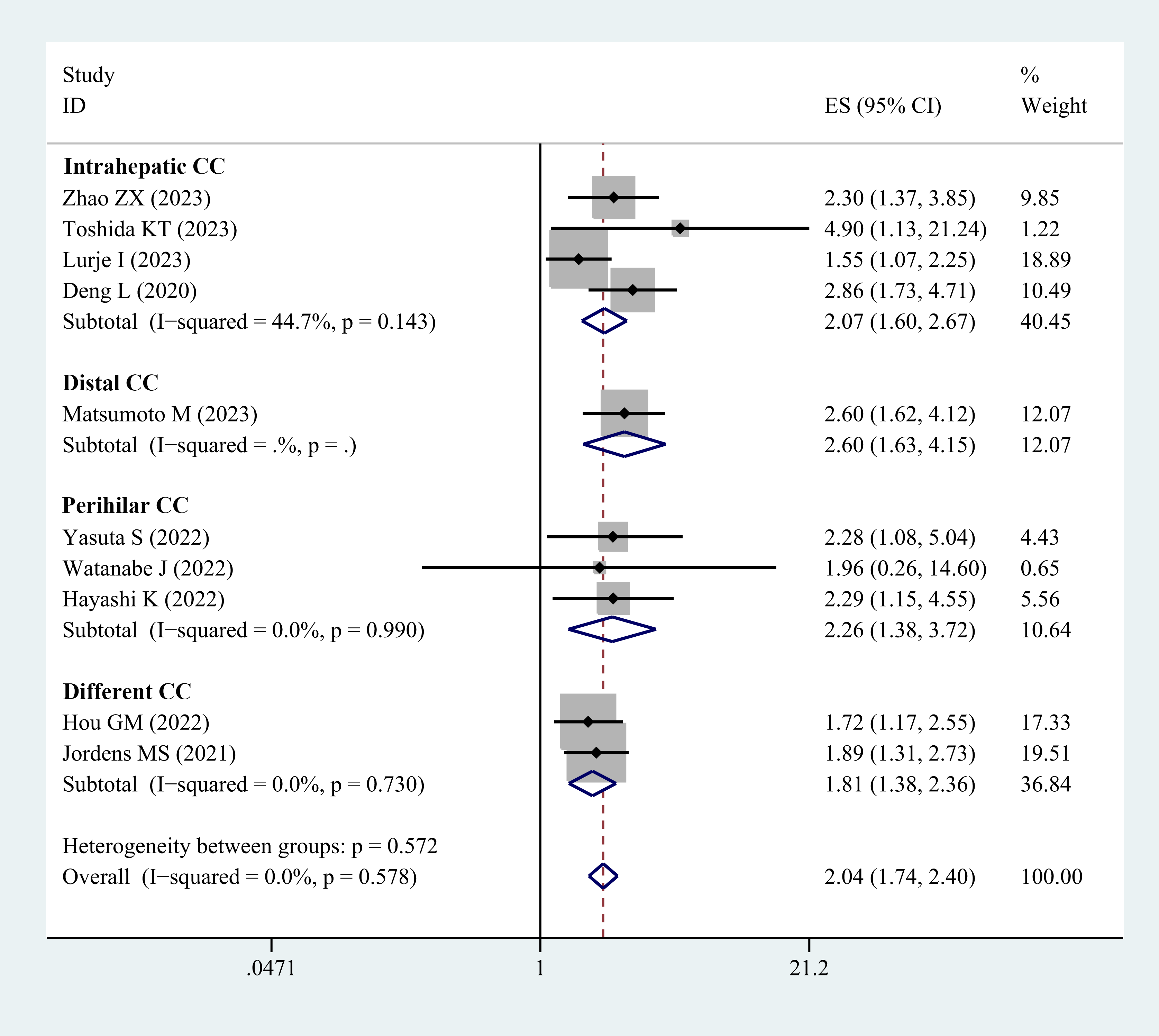

Supplement: Supplementary Figure 4 — Forest plot of studies evaluating hazard ratios of sarcopenia and the overall survival of cholangiocarcinoma (unadjusted hazard ratio values). [file Image_4.tif]

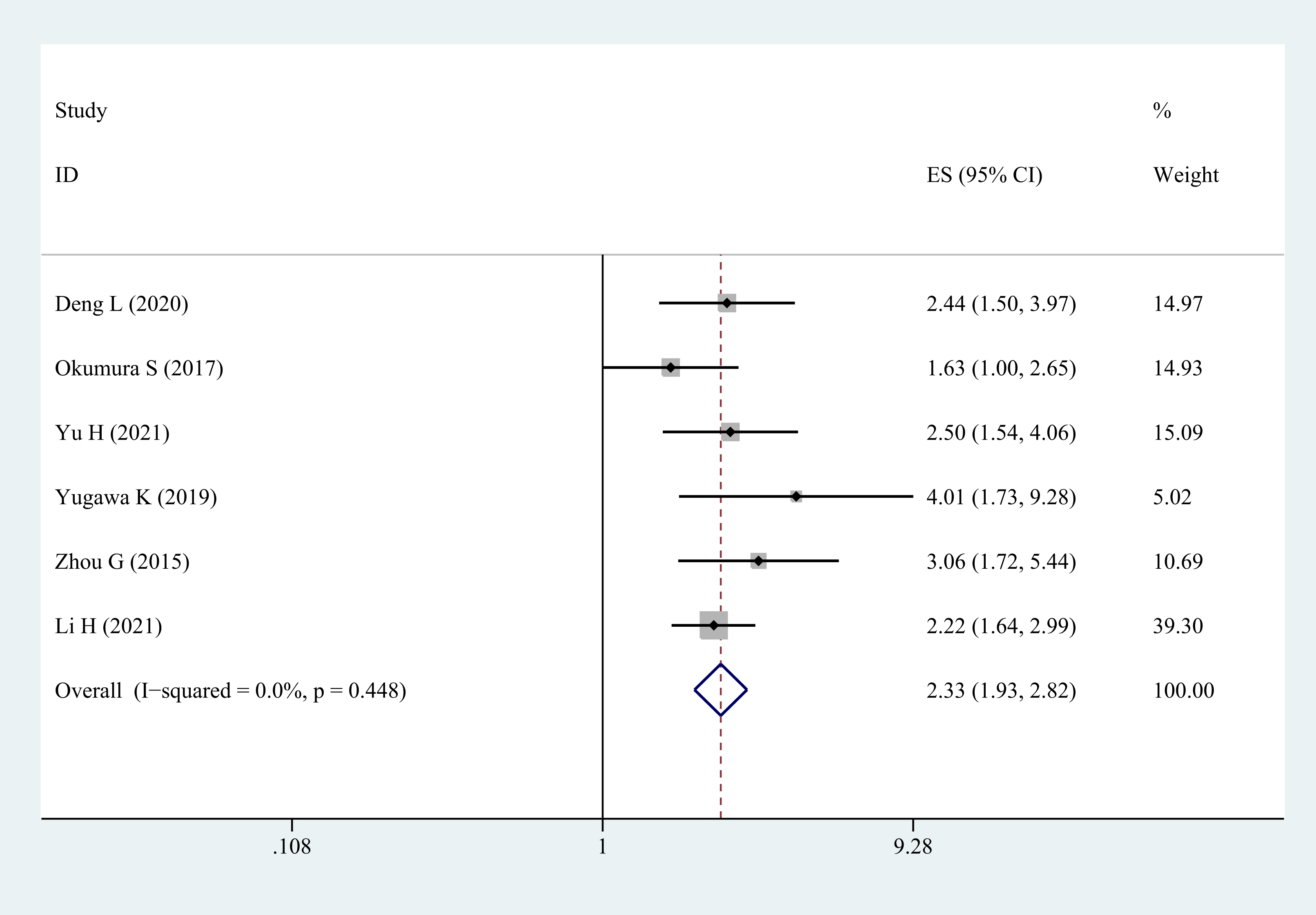

Supplement: Supplementary Figure 5 — Forest plot of studies evaluating hazard ratios of sarcopenia and the Recurrence-Free survival of cholangiocarcinoma (adjusted hazard ratio values). [file Image_5.tif]

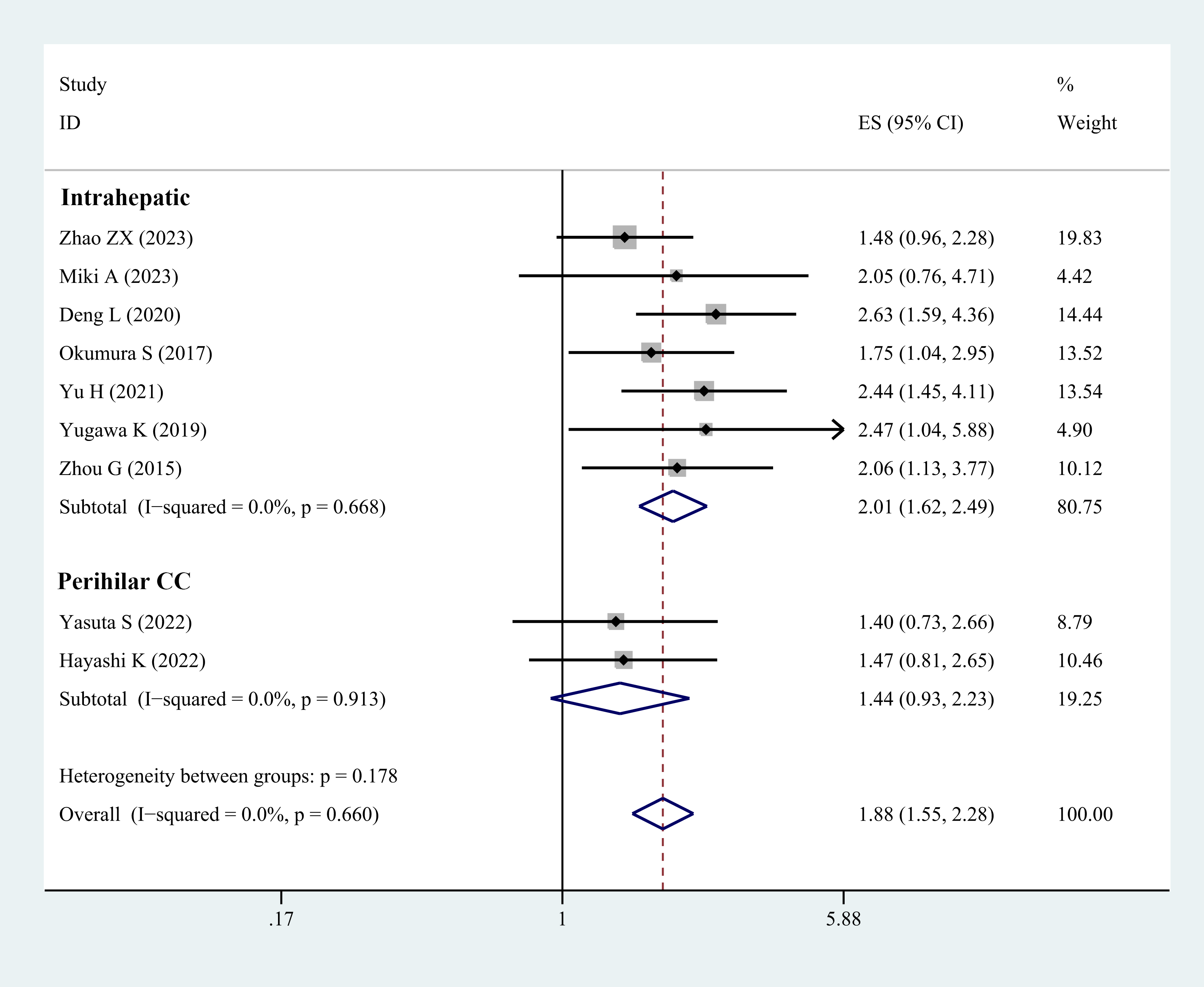

Supplement: Supplementary Figure 6 — Forest plot of studies evaluating hazard ratios of sarcopenia and the Recurrence-Free survival of cholangiocarcinoma (unadjusted hazard ratio values). [file Image_6.tif]

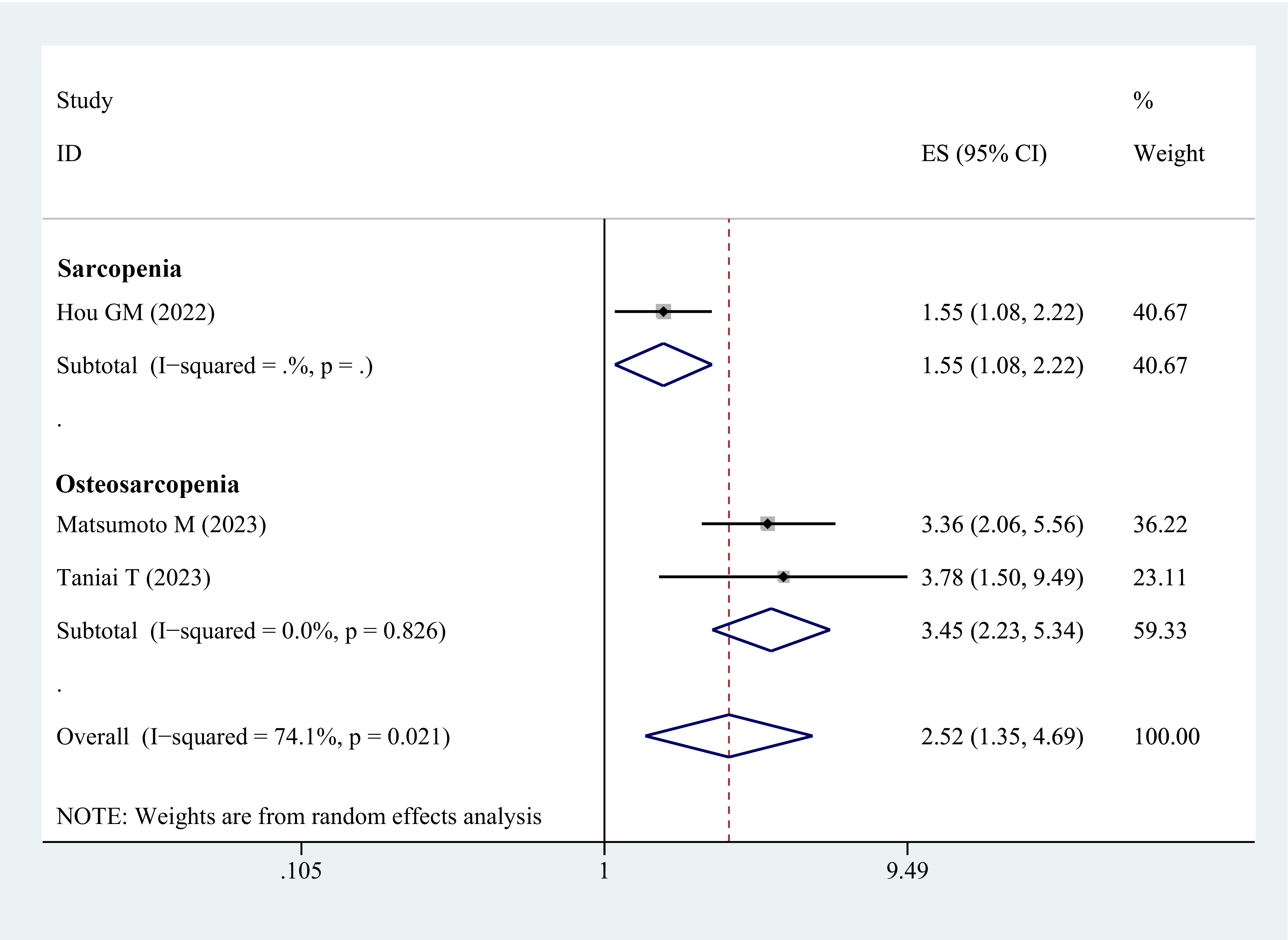

Supplement: Supplementary Figure 7 — Forest plot of studies evaluating hazard ratios of sarcopenia and the Disease-Free survival of cholangiocarcinoma (adjusted hazard ratio values). [file Image_7.tif]

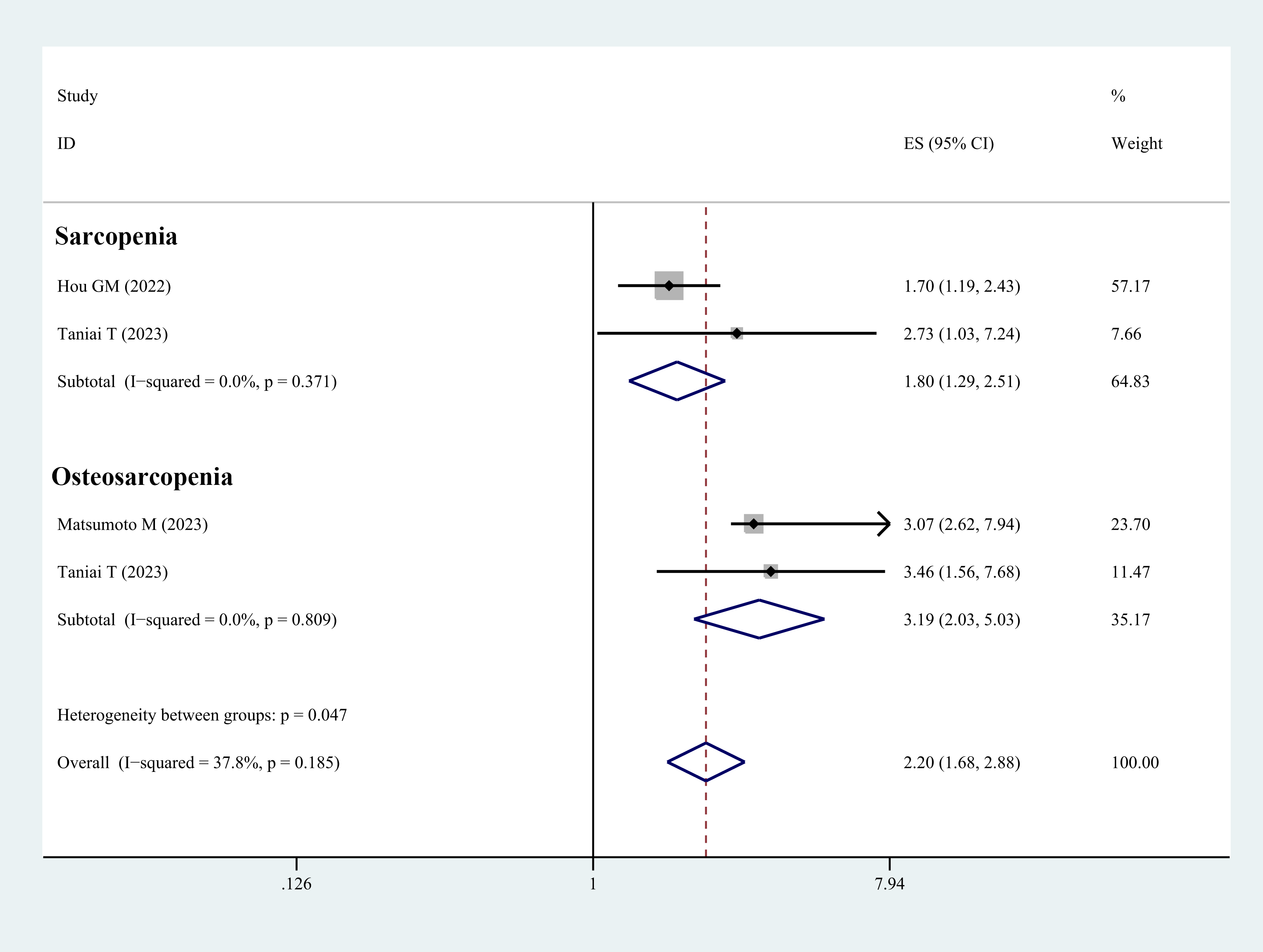

Supplement: Supplementary Figure 8 — Forest plot of studies evaluating hazard ratios of sarcopenia and the Disease-Free survival of cholangiocarcinoma (unadjusted hazard ratio values). [file Image_8.tif]

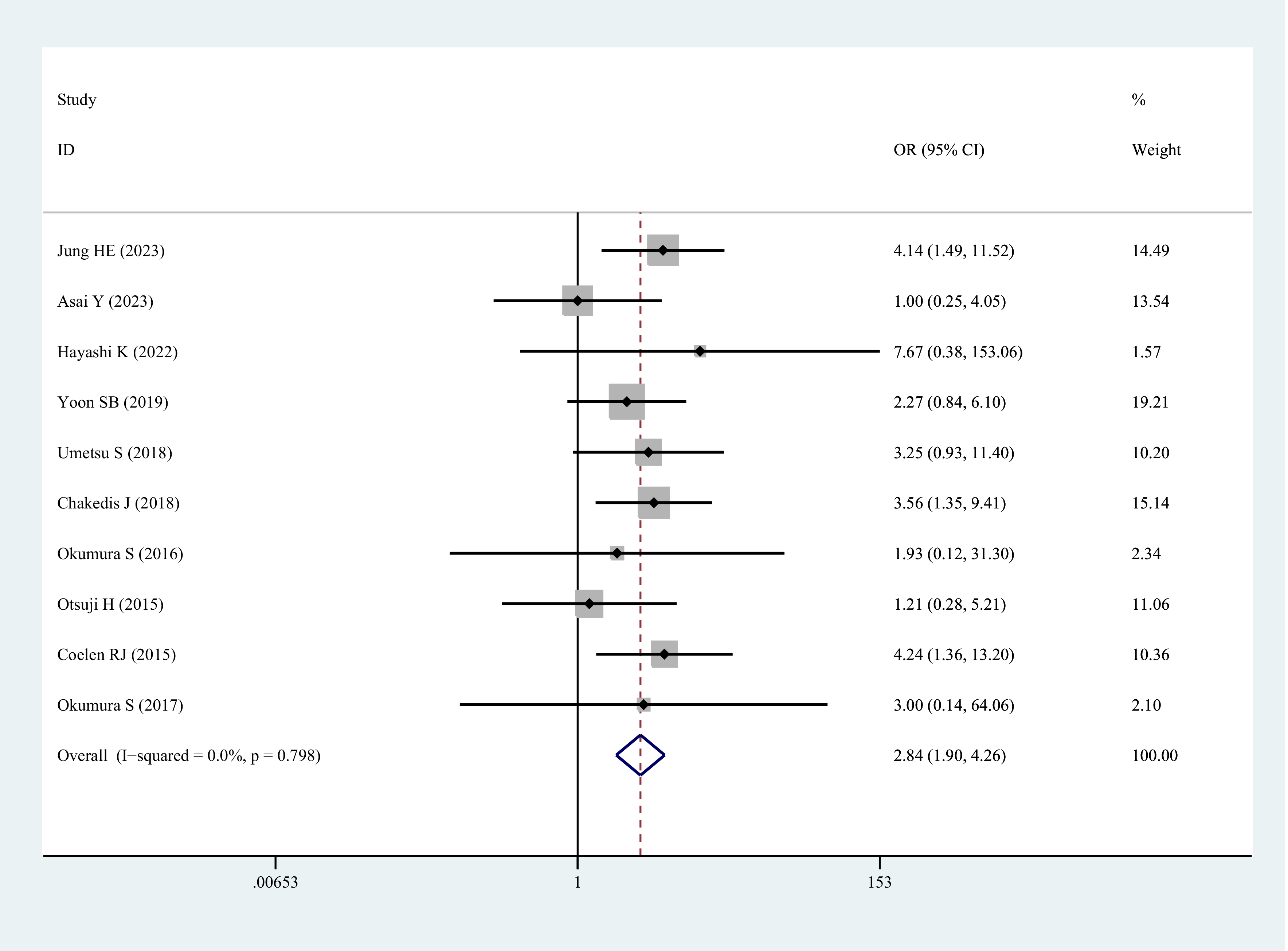

Supplement: Supplementary Figure 9 — Forest plot of odds ratios for mortality associated with surgery in sarcopenic patients compared with non-sarcopenic patients with cholangiocarcinoma. [file Image_9.tif]

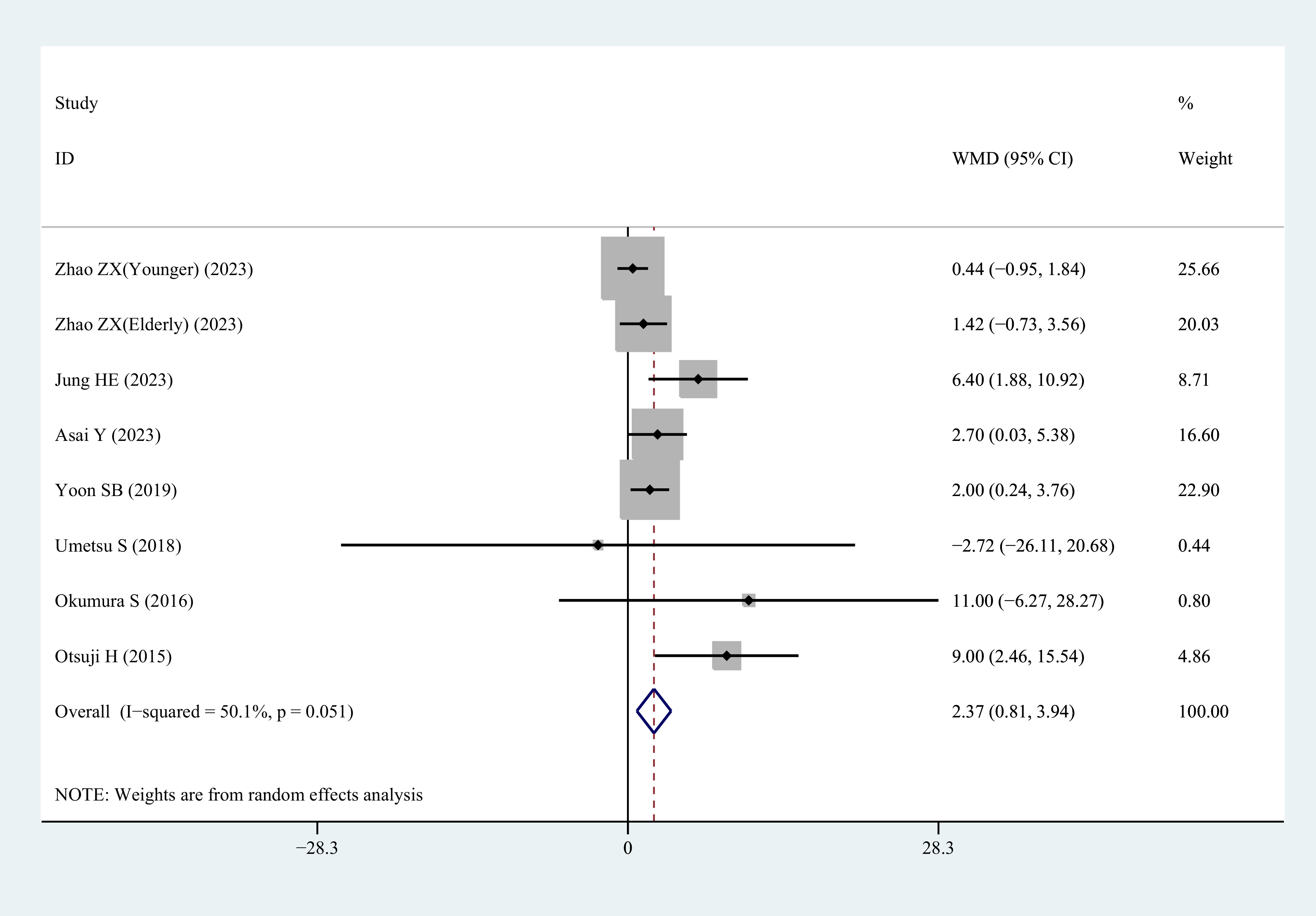

Supplement: Supplementary Figure 10 — The length of hospital stay between sarcopenic and nonsarcopenic patients with cholangiocarcinoma. [file Image_10.tif]
