# Supplementary material for: Prevalence and predictive value of sarcopenia in surgically treated cholangiocarcinoma: a comprehensive review and meta-analysis
Source: Front Oncol. 2024 Mar 19;14:1363843. doi: 10.3389/fonc.2024.1363843 (PMC10989063; doi:10.3389/fonc.2024.1363843)
Supplement: Supplementary file 11 [file Table_1.docx]

**Supplemental Material**

**Table S1. Search strategies in each database---2023.11.13**

| **Database** | **Search strategies** | **Results** |
| --- | --- | --- |
| **PubMed** | #1 "Sarcopenia"[Mesh]) OR (sarcopenia[Title/Abstract] OR sarcopenic[Title/Abstract] OR muscle mass[Title/Abstract] OR muscle strength[Title/Abstract] OR hand strength[Title/Abstract] OR grip strength[Title/Abstract] OR muscle atrophy[Title/Abstract] OR muscle wasting[Title/Abstract])---87,375  #2 "Cholangiocarcinoma"[Mesh]) OR (cholangiocarcinoma[Title/Abstract] OR extrahepatic cholangiocarcinoma[Title/Abstract] OR klatskin tumour[Title/Abstract] OR proximal bile duct cancer[Title/Abstract]---20,297  #3 #1 AND #2---56 | 56 |
| **Web of**  **Science** | #1 ((TS=(sarcopenia)) OR TI=(sarcopenia OR sarcopenic OR muscle mass OR muscle strength OR hand strength OR grip strength OR muscle atrophy OR muscle wasting)) OR AB=(sarcopenia OR sarcopenic OR muscle mass OR muscle strength OR hand strength OR grip strength OR muscle atrophy OR muscle wasting) ---109,594  #2 ((TS=(cholangiocarcinoma)) OR TI=(cholangiocarcinoma OR extrahepatic cholangiocarcinoma OR klatskin tumour OR proximal bile duct cancer)) OR AB=(cholangiocarcinoma OR extrahepatic cholangiocarcinoma OR klatskin tumour OR proximal bile duct cancer) ---16,219  #3 #1 AND #2---76 | 76 |
| **Embase** | #1 'sarcopenia'/exp OR sarcopenia:ti,ab,kw OR sarcopenic:ti,ab,kw OR 'muscle mass':ti,ab,kw OR 'muscle strength':ti,ab,kw OR 'hand strength':ti,ab,kw OR 'grip strength':ti,ab,kw OR 'muscle atrophy':ti,ab,kw OR 'muscle wasting':ti,ab,kw---126,673  #2 'cholangiocarcinoma'/exp OR cholangiocarcinoma:ti,ab,kw OR 'extrahepatic cholangiocarcinoma':ti,ab,kw OR 'klatskin tumour':ti,ab,kw OR 'proximal bile duct cancer':ti,ab,kw---42,843  #3 #1 AND #2---147 | 147 |
| **Cochrane library** | #1 MeSH descriptor: [Sarcopenia] explode all trees---854  #2 (sarcopenia OR sarcopenic OR muscle mass OR muscle strength OR hand strength OR grip strength OR muscle atrophy OR muscle wasting):ti,ab,kw (Word variations have been searched)---42,531  #3 #1 OR #2---42,531  #4 MeSH descriptor: [cholangiocarcinoma] explode all trees---328  #5 (cholangiocarcinoma OR extrahepatic cholangiocarcinoma OR klatskin tumour OR proximal bile duct cancer):ti,ab,kw (Word variations have been searched)---971  #6 #4 OR #5---973  #7 #3 AND #6---3 | 3 |
| **CNKI** | #1 (SU=sarcopenia) OR (TKA=sarcopenia OR sarcopenic OR muscle mass OR muscle strength OR hand strength OR grip strength OR muscle atrophy OR muscle wasting) ---17,181  #2 (SU=cholangiocarcinoma) OR (TKA=cholangiocarcinoma OR extrahepatic cholangiocarcinoma OR klatskin tumour OR proximal bile duct cancer)---19,102  #3 #1 AND #2---31 | 31 |
