# Supplementary material for: Prevalence and predictive value of sarcopenia in surgically treated cholangiocarcinoma: a comprehensive review and meta-analysis
Source: Front Oncol. 2024 Mar 19;14:1363843. doi: 10.3389/fonc.2024.1363843 (PMC10989063; doi:10.3389/fonc.2024.1363843)
Supplement: Supplementary file 12 [file Table_2.docx]

Supplementary Table 2 Characteristics of studies included in this meta-analysis

| Author | Year | Country | Study design | Age(years) | Pathological diagnosis | Assessment method  to sarcopenia | Cut-off values | Total participates （Male） | Outcome | Patients with  sarcopenia | NOS |
| --- | --- | --- | --- | --- | --- | --- | --- | --- | --- | --- | --- |
| Mir O^35^ | 2012 | France | Retrospective | 63 (41–83) | Different CC | SMI | Female:38.9 cm²/m² Male:55.4 cm²/m² | 28(19) | OS,Prevalence | 10 | 7 |
| Coelen RJ^22^ | 2015 | The Netherlands | Retrospective | Normal skeletal muscle mass ：62 ± 9 Low skeletal muscle mass ：61 ±11 | Perihilar extrahepatic CC | SMI | Female:39.1 cm²/m² Male:46.8 cm²/m² | 100(64） | RFS, DFS, OS, Complication, Sepsis, Prevalence, Mortality | 42 | 7 |
| Otsuji H^38^ | 2015 | Japan | Retrospective | Non-sarcopenia:67 ± 10 Sarcopenia:69 ± 9 | Distal extrahepatic CC | TPA | Female:396 mm²/m² Male:580 mm²/m² | 256(162) | Complication, Sepsis, Prevalence, Mortality, Length of stay | 84 | 7 |
| Zhou G^51^ | 2015 | China | Retrospective | 61 (47-81) | Intrahepatic CC | SMI | Female:41.10 cm²/m² Male:43.75 cm²/m² | 67(22） | OS, Prevalence, RFS | 33 | 8 |
| Okumura S^36^ | 2016 | Japan | Retrospective | Low PMI:69.8 ± 8.1 Normal PMI:65.4 ± 10.1 | Distal extrahepatic CC | PMI | Female:3.98 cm²/m² Male:6.15 cm²/m² | 207(111) | Complication, Sepsis, Prevalence, Mortality, Length of stay, RFS, OS | 71 | 7 |
| Okumura S^37^ | 2017 | Japan | Retrospective | 68 (61–73) | Intrahepatic CC | SMI | Female:41.2 cm²/m²  Male:52.5 cm²/m² | 109(67） | Complication, Prevalence， Mortality, OS, RFS | 67 | 7 |
| Chakedis J^21^ | 2018 | USA | Retrospective | 66 (50–82) | Different CC | PMI | Female: non-obese 4.21cm²/m²  obese 5.16 cm²/m² Male: non-obese 6.25 cm²/m² obese 7.32 cm²/m² | 117(52） | OS, Complication, Prevalence, Mortality | 41 | 8 |
| Umetsu S^42^ | 2018 | Japan | Retrospective | 72(31–81) | Distal extrahepatic CC | PMI | Female:3.54 cm²/m² Male:5.93 cm²/m² | 65(47) | Complication, Prevalence, Mortality, OS, Length of stay | 48 | 6 |
| Hahn F^24^ | 2019 | Germany | Retrospective | 66.0 (57‐73) | Intrahepatic CC | PMI | The resected subgroup:Female:5.1 cm²/m²  Male:5.7 cm²/m² The non‐resected subgroup:Female:4.8 cm²/m²  Male:5.5 cm²/m² | 293(176) | Prevalence, OS | 186 | 8/A |
| Kitano Y^29^ | 2019 | Japan | Retrospective | Sarcopenia:71 (48–81) Non-sarcopenia: 69 (19–88) | Distal extrahepatic CC | SMI | Female:41 cm²/m² Male:43 cm²/m² with BMI＜25  53 cm²/m² with BMI ≥25 | 110(75) | Complication, Prevalence, OS | 31 | 7 |
| van Vugt JLA^43^ | 2019 | The Netherlands | Retrospective | 66 (57–74) | Perihilar extrahepatic CC | SMI | Female:39.1 cm²/m² Male:46.8 cm²/m² | 233(140) | OS, Prevalence | 103 | 7 |
| Yoon SB^46^ | 2019 | Korea | Retrospective | 66.2 ± 9.6 | Different CC | SMI | Female:43.1 cm²/m² for Male:50.0 cm²/m² | 371(224) | OS, Complication, Prevalence, Mortality, Length of stay | 185 | 8 |
| Yugawa K^48^ | 2019 | Japan | Retrospective | High PMI:60 (39-82) Low PMI: 69 (53-87) | Intrahepatic CC | PMI | Female:18.1 cm² Male:34.6 cm² | 61(42) | Prevalence, RFS, OS | 30 | 6 |
| Deng L^23^ | 2020 | China | Prospective | 65 (40-87) | Intrahepatic CC | PMI | Female:6.04 cm²/m² Male:8.60 cm²/m² | 121(52) | Prevalence, OS, RFS | 53 | 8 |
| Zhang JX^49^ | 2020 | China | Retrospective | 56.4±22.9 | Perihilar extrahepatic CC | SMI | Female:35.14 cm²/m² Male:46.95 cm²/m² | 104(56) | Complication, Prevalence | 61 | 7 |
| Jordens MS^27^ | 2021 | Germany | Retrospective | 68.5 (41–89) | Different CC | SMI | SMI:54.26 cm²/m² PMI:1.685 cm²/m² | 76(37) | OS | NA | 7 |
| Li H^30^ | 2021 | China | Retrospective | Discovery cohort:58 (49, 64) Validation cohort: 59 (50, 67) | Intrahepatic CC | SMI | Female:37.8 cm²/m² Male:42.6 cm²/m² | 460(223) | OS, Prevalence | 281 | 9 |
| Tamura S^39^ | 2021 | Japan | Retrospective | 72 (39-85) | Distal extrahepatic CC | SMI | Female:36 cm²/m² Male:55 cm²/m² | 111(86) | RFS, DFS, OS, Prevalence | 89 | 8 |
| Yu H^47^ | 2021 | China | Prospective | 64.94 ± 10.12 | Intrahepatic CC | PMI | Female:6.0 cm²/m² Male:8.6 cm²/m² | 116(51) | Prevalence, OS, RFS | 49 | 8 |
| Ardito F^19^ | 2022 | Italy | Retrospective | 66 (25–79) | Intrahepatic CC | SMI | Female:41.2 cm²/m² Male:52.5 cm²/m² | 30(20) | Complication, Prevalence | 15 | 6 |
| Hayashi K^25^ | 2022 | Japan | Retrospective | Preoperative skeletal muscle no-change:72 (65–77) Preoperative skeletal muscle wasting:73 (65–77) | Perihilar CC | PMI | Female:3.92 cm²/m² Male:6.36 cm²/m² | 89(55) | Complication, Prevalence, Mortality, OS, RFS | 25 | 7 |
| Hou GM^26^ | 2022 | China | Retrospective | NA | Different CC | PMI | Female:4.05 cm²/m² Male:5.42 cm²/m² | 153(128) | RFS, DFS, OS, Prevalence | 77 | 7 |
| Lurje I^31^ | 2022 | Germany | Retrospective | Intrahepatic CC:65±11.4 Perihilar CC:66±10.4 | Perihilar and intrahepatic CC | SMI | BMI<25 kg/m²:Female:41 cm²/m²  Male:43 cm²/m² BMI≥25 kg/m²:Female:53 cm²/m²  Male:53 cm²/m² | 189(108) | RFS, DFS, OS, Prevalence | 68 | 7 |
| Watanabe J^44^ | 2022 | Japan | Retrospective | 68.5(41-89) | Perihilar CC | PMI | Female:3.92 cm²/m² Male:6.36 cm²/m² | 58(42) | OS | NA | 6 |
| Yasuta S^45^ | 2022 | Japan | Retrospective | 70 (39–81) | Perihilar CC | SMI | Female:41cm²/m²  Male :43cm²/m²with BMI＜25kg/m²  Male :53cm²/m²with BMI≥25kg/m² | 56(38) | Prevalence, OS, RFS | 26 | 7 |
| Asai Y^20^ | 2023 | Japan | Retrospective | PMI: Normal 68 (61-73) Low:72 (65-77) | Perihilar CC | PMI | Female:18.1 cm²/m² Male:34.6 cm²/m² | 456(308) | OS, Complication, Prevalence, Mortality, Length of stay | 151 | 6 |
| Jung HE^28^ | 2023 | Korea | Retrospective | 65.6 ± 9.0 | Different CC | PMI | Female:3.39 cm²/m² Male:6.74 cm²/m² | 317(202) | RFS, DFS, OS, Complication, Sepsis, Prevalence, Mortality, Length of stay | 149 | 6 |
| Lurje I^32^ | 2023 | Germany | Retrospective | 65.0 (23.0–83.0) | Intrahepatic CC | SMI | Female:38.5 cm²/m² Male:52.4 cm²/m² | 173(86) | RFS, DFS, OS, Prevalence | 30 | 7 |
| Matsumoto M^33^ | 2023 | Japan | Retrospective | 71 (35–87) | Distal extrahepatic CC | PMI | Female:13.3 cm² Male:26.4 cm² | 138(94) | Prevalence, DFS, OS | 30 | 7 |
| Miki A^34^ | 2023 | Japan | Retrospective | Normal PMI:64.9 ± 9.7 Low PMI:70.3 ± 7.2 | Intrahepatic CC | PMI | Female:3.92 cm²/m² Male:6.36 cm²/m² | 71(46) | Prevalence, OS, RFS | 61 | 6 |
| Taniai T^40^ | 2023 | Japan | Retrospective | 63 (55–68) | Intrahepatic CC | PMI | Female:14.94 cm² Male:31.47 cm² | 41(21) | Prevalence, DFS | 28 | 6 |
| Toshida KT^41^ | 2023 | Japan | Retrospective | Normal SMI:65 (33–87) Low SMI:69 (41–87) | Intrahepatic CC | SMI | Female:<38 cm²/m² Male:<42 cm²/m² | 95(63) | Prevalence, OS | 16 | 7 |
| Zhao ZX^50^ | 2023 | China | Retrospective | Elder:70 (67–75) Younger:58 (51–62) | Intrahepatic CC | SMI | Female:39.9 cm²/m² Male:53.5 cm²/m² | 302(151) | RFS, DFS, OS, Complication, Prevalence, Length of stay | 193 | 8 |

CC, cholangiocarcinoma; PMI, Psoas Muscle Index; SMI, Skeletal Muscle Index; NA, not applicable; RFS, Recurrence-Free Survival; DFS, Disease-Free Survival; OS, overall survival; NOS, Newcastle-Ottawa Scale.
