# Supplementary material for: Prevalence and predictive value of sarcopenia in surgically treated cholangiocarcinoma: a comprehensive review and meta-analysis
Source: Front Oncol. 2024 Mar 19;14:1363843. doi: 10.3389/fonc.2024.1363843 (PMC10989063; doi:10.3389/fonc.2024.1363843)
Supplement: Supplementary file 13 [file Table_3.docx]

Supplementary Table 3 Subgroup analyses in terms of overall survival, disease-free survival and Recurrence-free survival (unadjusted HR).

| Characteristics | Overall survival | | | | Disease-free survival | | | | Recurrence-free survival | | | |
| --- | --- | --- | --- | --- | --- | --- | --- | --- | --- | --- | --- | --- |
|  | n | HR (95%CI) | P | Heterogeneity | n | HR (95%CI) | P | Heterogeneity | n | HR (95%CI) | P | Heterogeneity |
| Anatomical classification of CC | 10 | 2.04(1.74,2.40) | <0.001 | 0.00% | 3 | 2.20(1.68,2.88) | 0.004 | 74.10% | 9 | 1.88(1.55,2.28) | <0.001 | 0.00% |
| Intrahepatic CC | 4 | 2.07(1.60,2.67) | <0.001 | 44.70% |  |  |  |  | 7 | 2.01(1.62,2.49) | <0.001 | 0.00% |
| Perihilar CC | 3 | 2.26(1.38,3.72) | <0.001 | 0.00% |  |  |  |  | 2 | 1.44(1.93,2.23) | <0.001 | 0.00% |
| Distal CC | 1 |  | NA |  |  |  |  |  |  |  |  |  |
| Different CC | 2 | 1.81(1.38,2.36) | <0.001 | 0.00% |  |  |  |  |  |  |  |  |
| Ethnicity |  |  |  |  |  |  |  |  |  |  |  |  |
| Asian | 8 | 2.28(1.86,2.80) | <0.001 | 0.00% |  |  |  |  |  |  |  |  |
| Caucasian | 2 | 1.71(1.32,2.22) | <0.001 | 0.00% |  |  |  |  |  |  |  |  |
| Test for sarcopenia |  |  |  |  |  |  |  |  |  |  |  |  |
| PMI | 6 | 2.12(1.74,2.59) | <0.001 | 0.00% |  |  |  |  | 6 | 2.09(1.64,2.67) | <0.001 | 0.00% |
| SMI | 4 | 1.90(1.44,2.50) | <0.001 | 14.50% |  |  |  |  | 3 | 1.59(1.17,2.17) | 0.003 | 0.00% |
| Adjuvant chemotherapy |  |  |  |  |  |  |  |  |  |  |  |  |
| Yes | 0 |  | NA |  |  |  |  |  | 2 | 2.06(1.24,3.40) | 0.005 | 0.00% |
| No | 4 | 2.11(1.71,2.61) | <0.001 | 16.40% |  |  |  |  | 2 | 2.54(1.77,3.64) | <0.001 | 0.00% |
| Any | 6 | 1.95(1.51,2.51) | <0.001 | 0.00% |  |  |  |  | 5 | 1.59(1.24,2.05) | <0.001 | 0.00% |

CC, cholangiocarcinoma; PMI, Psoas Muscle Index; SMI, Skeletal Muscle Index; NA, not applicable.
